# Single-cell BCR and transcriptome analysis reveals peripheral immune signatures in patients with thyroid-associated ophthalmopathy

**DOI:** 10.18632/aging.205814

**Published:** 2024-05-09

**Authors:** Qian Li, Ningyu An, Cheng Liu, Yungang Ding, Cuixia Yang, Xiumei Ma, Wei Yang, Junfeng Piao, Jinyan Zhu, Junxiu Liu

**Affiliations:** 1 Department of Ophthalmology, Peoples’ Hospital of Ningxia Hui Autonomous Region, The Third Affiliated Clinical College of Ningxia Medical University, Yinchuan, Ningxia, P.R. China; 2 Medical Science Research Institution of Ningxia Hui Autonomous Region, Medical Sci-Tech Research Center of Ningxia Medical University, Yinchuan, Ningxia, P.R. China; 3 Affiliated Eye Hospital of Shandong University of Traditional Chinese Medicine, Jinan, Shandong, P.R. China; 4 Department of Ophthalmology, Guro Hospital, Korea University College of Medicine, Guro-gu, Seoul 152–703, South Korea

**Keywords:** scRNA-seq, scBCR-seq, thyroid associated ophthalmology, Bregs

## Abstract

Thyroid-associated ophthalmopathy (TAO) is the most prevalent orbital disease in adults caused by an autoimmune disorder, which can lead to disfigurement and vision impairment. Developing effective treatments for this condition presents challenges due to our limited understanding of its underlying immune aberrations. In this study, we profiled the immune components in the peripheral blood of patients with TAO as well as healthy individuals, utilizing single-cell RNA sequencing and B-cell receptor repertoires (BCR) analysis. We observed a significant reduction in the proportions of regulatory B cells (Bregs) and type 2 conventional dendritic cells (DCs) in patients with TAO during the active phase. Conversely, there was a significant increase in the proportion of type 1 DCs. Further analysis of cell differentiation trajectory revealed potential impairment in the transition of B cells towards Breg phenotype during the active phase of TAO. Besides, the activation process of TAO appeared to involve inflammation and immune dysfunction, as indicated by the dynamic changes in the activities of key regulators. The abnormalities in the peripheral immune system, such as the reduced capacity of Bregs to suppress inflammation, were primarily driven by the enhanced interaction among Breg, DCs, and monocytes (i.e., CD22-PTPRC and BTLA-TNFRSF14). Collectively, our findings offer a comprehensive insight into the molecular regulation and cellular reconfiguration during the active phase of TAO at the single-cell level, in order to explore the pathogenesis of TAO and provide new ideas for the future treatment of TAO.

## INTRODUCTION

Thyroid-associated ophthalmopathy (TAO) is an orbit-specific autoimmune disease, which cause disfigurement and has the potential to lead to blindness [1]. At present, the cause of TAO is not clear. TAO can be combined with hyperthyroidism and hypothyroidism, and the number of patients with normal thyroid function is increasing year by year. It is a kind of organ-specific autoimmune disease which is related to thyroid function and relatively independent. With characteristic ocular signs, the common symptom of TAO is eyelid retraction, with or without exophthalmos, which accounts for 70-80% of all TAO. It is the main extrathyroidal manifestation of Graves’ disease (GD) and affects approximately 20–50% of GD patients [2]. Due to our limited understanding of its potential immune aberrations, developing effective treatments presents a challenge. Patients with TAO experience significant challenges due to a range of clinical manifestations that vary during different phases of the disease. The initial active phase lasting typically 1-3 years, is characterized by inflammation of the orbital tissues [3]. During this phase, patients may exhibit eyelid swelling, conjunctival congestion, chemosis, proptosis, and strabismus, which result from the swelling of the orbital fat and extraocular muscles (EOM). The expansion of orbital volume exerts pressure on the optic nerve, leading to compressive optic neuropathy [4]. Subsequently, the disease enters a quiescent phase, during which it becomes inactive. In this phase, the pathologic changes are dominated by the development of adipose tissue and myofibroblasts in the periocular area, representing the final stage of the inflammatory cascade [5]. Various manifestations can persist, ranging from mild eyelid retraction and proptosis to moderate diplopia and visual impairment. As a result, TAO significantly impacts patients' quality of life and imposes a substantial burden on public health, both in terms of direct and indirect costs [6, 7]. This study used single-cell RNA sequencing and B cell receptor profile (BCR) analysis to analyze the immune components in peripheral blood of patients and healthy individuals with TAO, providing comprehensive insights into the molecular regulation and cell remodeling of the active phase of TAO at the single-cell level, in order to explore the pathogenesis of TAO and provide new ideas for the future treatment of TAO.

The lack of effective treatments that can alter the natural progression has made managing TAO challenging, primarily due to our poor limited understanding of its underlying causes [8]. Glucocorticoids, the main treatment for active TAO, are often successful in reducing inflammation [9]. However, less than 70% of patients benefit from this approach, and 10%-20% experience a relapse after discontinuing the medication [2]. External beam radiotherapy is recommended to address the enlargement of extraocular muscles, either alone or in combination with steroid [10]. Nonetheless, the application of both therapies is hindered by potentially serious side effects, including the exacerbation of glucose intolerance, hypertension, psychiatric diseases, and radiation induced retinopathy [11, 12]. Despite the availability of various therapeutic approaches, rehabilitative surgeries, such as orbital decompression for patients with urgent compressive optic neuropathy or exposure corneal ulcer, are inevitably required when the disease become inactive [8].

It is crucial to enhance our understanding of the pathogenesis of TAO. The development and progression of TAO is a cascade of autoimmune events involving a complex network of interactions between immune cells and orbital fibroblasts (OFs) [3]. Thyroid-stimulating immunoglobulins (TSIs), which are antibodies that target the thyroid-stimulating hormone receptor (TSHR), binds to TSHR and recruit antigen presenting cells (APC) such as B cells, dendritic cells (DCs), and monocytes [13]. B cells interact with T cells through major-histocompatibility-complex (MHC) molecules, costimulatory molecules (CD80 and CD86), and CD40 [14]. Upon antigenic stimulation, CD4^+^ T cells differentiate into different subsets such as Th1 cells, Th2 cells, Th17 cells, and produce cytokines like interferon-γ (IFN-γ), tumor necrosis factor-α (TNF-α), and interleukin-1β (IL-1β) [15, 16]. These cytokines released by CD4^+^ T cells activate and continuously stimulate OFs [17, 18]. As the target cells in TAO, OFs secrete abundant hyaluronan and differentiate into adipocytes and myofibroblasts [19], which subsequently lead to inflammation, glycosaminoglycan accumulation, adipogenesis, and myofibrogenesis of orbital tissues [20]. Additionally, B cells further differentiate into plasma cells that produce antibodies and spontaneously secrete TSIs after interaction with T cells through CD40/CD40 ligand [21].

Recent advancements in single-cell RNA sequencing (scRNA-seq) have provided a valuable tool for examining specific cell populations and elucidating the intercellular interactions at the single-cell resolution. Current studies utilizing scRNA-seq to investigate the pathology of TAO have primarily focused on studying the local orbits tissues. These studies have successfully identified novel T cell subsets and provided further insights into the adipogenic and profibrotic characteristics of OFs [5, 16, 22–24]. However, the extent to which the imbalance of immune regulation in circulating lymphocytes, such as B cells and myeloid cells, contributes to TAO remains largely unknown. Additionally, it is unclear whether there are differences in the underlying molecular mechanisms between active and inactive phases of TAO.

In this study, we employed scRNA-seq and single-cell BCR sequencing (scBCR-seq) techniques to investigate the variations in cell compositions and BCR repertoires among individuals with active TAO, inactive TAO, and NCs. Our analysis unveiled significant changes in the composition of immune cells, with a particular focus on B cells, dendritic cells, and monocytes. Additionally, we delved into cellular processes, signaling pathways, and cell-cell interactions to elucidate the mechanisms driving the re-configuration of peripheral blood mononuclear cells (PBMCs) during active TAO. Furthermore, the results of this research offer valuable resources that can potentially lead to the development of novel therapeutic strategy for TAO in the future.

## MATERIALS AND METHODS

### Study design

Blood samples from 6 TAO patients and 3 NCs were used to conduct scRNA-seq and scBCR-seq. The baseline information of these samples was listed in Table 1. 15 inactive TAO patients, 7 active TAO patients, and 10 NCs were used for flowcytometry analysis. The basic characteristics of these samples were listed in Table 2. All patients were diagnosed based on Bartley criteria [25]. The patients with other autoimmune diseases, accepted glucocorticoid and/or immunosuppressor treatments were excluded. Healthy adults without autoimmune disease history or infection within the last 3 months were recruited as the NCs and were matched by age and sex. Disease severity was evaluated based on the NOSPECS classification [26].

**Table 1:** The baseline information of the samples used for scRNA-seq and scBCR-seq.

**Patients**	**Gender**	**Age (years)**	**CAS Score**	**NOSPECS Score**	**Disease condition**
P1	Female	53	4	3	Active
P2	Female	43	5	6	Active
P3	Male	62	4	4	Active
P4	Male	56	0	3	Inactive
P5	Female	38	1	3	Inactive
P6	Female	55	0	3	Inactive
NC1	Female	69	-	-	Normal
NC2	Female	58	-	-	Normal
NC3	Male	51	-	-	Normal

**Table 2:** The basic characteristics of the samples from normal controls (n=10), patients with inactive TAO (n=15), and patients with active TAO (n=7) during the flowcytometry analysis.

	**Normal controls**	**Active TAO**	**Inactive TAO**
Age (years)			
Mean	42.40	54.43	35.53
Range	31-58	36-78	19-57
Sex (no.)			
Male	6	4	5
Female	4	3	10
Biochemical characteristics			
FT3 (pmol/L)	-	5.27+2.00	4.90+1.46
FT4 (pmol/L)	-	16.50+7.26	14.12+6.04
TSH (IU/L)	-	1.83+2.59	3.25+8.10

### Peripheral blood monocytes (PBMCs) isolation

The PBMCs were isolated by using a density gradient centrifugation method. Briefly, peripheral blood samples were transferred to a 15mL centrifuge tube and diluted with 1xPBS of equal volume. Equal volume of human peripheral blood lymphocyte separation (Ficoll-Paque, P8900, Solarbio, China) was added to the diluted samples. After mixing, the samples were centrifuged at 700g at 4° C for 30min. The PBMCs located between 1xPBS and Ficoll layer were transferred to a new centrifuge tube, and 1xPBS was added to clean the cells. An appropriate volume (200ul) was taken into 1.5mL centrifuge tube, and a triploid volume (600ul) of erythrocyte lysate was added, gently upside down mixed and placed ice lysis for 8 min. After erythrocyte lysis, incubated at 4° C for 4~5min, centrifuged at 300g at 4° C for 10 min immediately after completion and removing the supernatant, and added 1 ml of re-suspension (precooled 1x PBS+0.04% BSA without calcium and magnesium) to fully re-suspended the cells. Then test with cell counter (Countstar Rigel S2), adjusted the concentration according to the test result. The target concentration was 700-1200 cell/μL, the cell activity was greater than 90%, and the agglomeration was less than 15%. Once the final cell concentration and activity were reached, the cells were placed on ice and 10X Genomics single cell transcriptome chip on-board experiment was carried out within 30 minutes.

### scRNA-seq library preparation and sequencing

We prepared single cell RNA-seq libraries with Chromium Next GEM Single Cell 3ʹ Reagent Kits v3.1 (1000268) on the Chromium. We prepared single cell suspensions from cultured cell lines and single cells were suspended in PBS containing 0.04% BSA. The cell suspension was loaded onto the Chromium Next GEM Chip G and ran the Chromium Controller to generate single-cell gel beads (Beckman Coulter-SPRI select Reagent Kit-B23318, USA) in the emulsion (GEMs) according to the manufacturer’s recommendation. Chromium Next GEM Chip K Single Cell Kit (48 rxns, 1000286) was used to capture cells. Captured cells were lysed and the released RNA was barcoded through reverse transcription in individual GEMs. Barcoded, full-length cDNA was generated and libraries were constructed according to the performer’s protocol. Chromium Next GEM Single Cell 5' Reagent Kits v2 (Dual Index) (16 rxns, PN1000263), Library Construction Kit (16 rxns, PN1000190) and Dual Index Plate TT Set A (96 rxns, PN3000431) were used to construct libraries. The quality of libraries was assessed by Agilent-High Sensitivity DNA Kit (5067-4626). Sequencing was performed on the Illumina NovaSeq 6000 with a sequencing depth of at least 50,000 reads per cell and 150 bp (PE150) paired-end reads (performed by Biomarker Technologies Corporation, Beijing, China).

### Processing raw data and assay from scRNA-seq of 10× genomics

We performed alignment to this amended reference using 10x cellranger 6.0.0, which employs the STAR sequence aligner [27]. The reference genome was the human genome GRCh38. Overall, 93007 cells passed the quality control (Agilent-High Sensitivity DNA Kit -5067-4626). To remove the cells with low quality, cells with gene number over 500 and the ratio of mitochondria lower than 0.20 were maintained, and genes with at least one feature count in more than three cells were used for the following analysis. We determined gene expression counts using unique molecular identifiers (UMIs) for each cell barcode-gene combination. Following alignment, we filtered cell barcodes to identify those which contain cells using the approach implemented in cellranger 3.0.2, and only these barcodes were considered for downstream analysis including clustering and cell type identification, differential expression analysis by Seurat (version 4.0.1).

### Clustering and cell type identification

We applied Louvain community detection [28] to the nearest neighbor graph constructed in PCA space to define a cluster partition. To infer cell types, we trained a neural network classifier to predict cell ontology classes given single-cell RNA-seq mRNA abundance profiles. First, we use singleR (4.0.4) which contains 7 data sets to automate the identification of cell types. In addition to these annotations, we manually added cell state annotations to SingleR to provide a level of granularity below cell ontology classes. After completing the annotations, we further corrected the cell types using the CellMarker database (http://bio-bigdata.hrbmu.edu.cn/CellMarker1.0/).

### Gene ontology enrichment analysis, cell cycle scoring

We used Enrichr [29] to perform gene set enrichment analysis against the Gene Ontology Biological Process 2018 version gene set collection. We also used the MSigDB Hallmark gene sets [30], for which we computed enrichment scores using Fisher’s exact test. In both cases, we corrected for multiple hypothesis testing using the Benjamini-Hochberg procedure. We estimated cell cycle activity by scoring the expression of a set of S phase–associated and G2/M phase–associated genes, as shown previously [31] and as implemented in Seurat 4.0.1 [28].

### Trajectory inference

We used the Monocle 2 (version 2.18.0) to place cells onto pseudotime trajectories (https://github.com/cole-trapnell-lab/monocle-release), which applies reversed graph embedding (RGE), a recently developed machine learning strategy, to accurately reconstruct complex single-cell trajectories. Monocle 2 requires the genes without a priori information that characterize the biological process, the number of cell fates or branch points in the trajectory, or the design of the experiment. Monocle 2 outperforms not only its previous version but also more recently developed methods, producing more accurate, robust trajectories. The Monocle 2 analysis is based on the following three steps. First, genetic screening: monocle looks for genes that change in “interesting” (not just noisy) ways, and uses those genes to construct the data. Second, lower dimensions: once the genes for cell sequencing are selected, Monocle reduces the dimensions of the data. Third, pseudotime cell analysis: by projecting expression data into a lower dimensional space, the differentiation locus between cells was constructed.

### ScBCR-seq library preparation and sequencing

### GEM generation and barcoding

GEMs are generated by combining barcoded Single Cell 5’ Gel Beads (Chromium Next GEM Single Cell 5' Reagent Kits v2 (Dual Index), 16 rxns, PN100026), a Master Mix with cells, and Partitioning Oil on a microfluidic chip. To achieve single cell resolution, the cells are delivered at a limiting dilution, such that the majority (~90–99%) of generated GEMs contains no cell, while the remainder largely contain a single cell. Immediately following generation of a GEM, the Single Cell 5’ Gel Bead is dissolved and any co-partitioned cell is lysed.

Upon dissolution of the Single Cell 5’ Gel Bead in a GEM, oligonucleotides containing (i) an Illumina® R1 sequence (read 1 sequencing primer), (ii) a 16 nt 10x Barcode, (iii) a 10 nt Unique Molecular Identifier (UMI), and (iv) a 13 nt template switch oligo (TSO) are released and mixed with cell lysate and a Master Mix that contains reverse transcription (RT) reagents and poly(dT) primers.

Incubation of the GEMs then produces barcoded, full-length cDNA from poly-adenylated mRNA. After incubation, the GEMs are broken and the pooled post GEM-RT reaction mixtures are recovered.

### GEM-RT clean up

The pooled post GEM-RT reaction mixture contains barcoded first-strand cDNA from poly-adenylated mRNA, as well as leftover biochemical reagents and primers. Silane magnetic beads are used to purify the cDNA from this mixture. Barcoded, full-length V(D)J segments can then be directly enriched from the purified post Silane magnetic beads which are used to remove leftover biochemical reagents and primers from the post GEM reaction mixture. Full-length, barcoded cDNA is then amplified by PCR to generate sufficient mass for library construction.

### cDNA amplification

Barcoded, full-length cDNA is amplified via PCR with primers against common 5’ and 3’ ends added during GEM-RT. This amplification reaction generates sufficient material to construct multiple libraries from B cell-enriched libraries and 5’ gene expression libraries.

### Target enrichment

Barcoded, full-length V(D)J segments are enriched from amplified cDNA via PCR amplification with primers specific to Ig constant regions prior to library construction.

### Enriched library construction

Enzymatic Fragmentation and Size Selection are used to generate variable length fragments that collectively span the V(D)J segments of the enriched Ig transcripts prior to library construction. R1 (read 1 primer sequence) is added to the molecules during GEM-RT incubation. P5 is added during Target Enrichment. P7, a sample index and R2 (read 2 primer sequence) are added during library construction via End Repair, A-tailing, Adaptor Ligation and PCR. The final libraries contain the P5 and P7 priming sites used in Illumina® bridge amplification.

### Sequencing libraries

The Single Cell V(D)J Reagent Kit (Chromium Single Cell Human TCR Amplification Kit, 16 rxns, PN1000252; Chromium Single Cell Human BCR Amplification Kit, 16 rxns, PN1000253) protocol produces V(D)J enriched and 5’ gene expression Illumina-ready sequencing libraries. A library comprises standard Illumina paired-end constructs which begin and end with P5 and P7. For V(D)J enriched libraries, Read 1 encodes the 16 bp 10x™ Barcode, 10 bp UMI, and 13 bp Switch Oligo, as well as the 5’ end of an enriched transcript. For 5’ gene expression libraries, Read 1 encodes the 16 bp 10x Barcode and 10 bp UMI. Due to Enzymatic Fragmentation, for both libraries Read 2 encodes a random internal fragment of the corresponding insert. Sample index sequences are incorporated as the i7 index read.

### 5’ gene expression data processing

### Processing raw data and assay

We performed alignment to this amended reference using 10x cellranger 6.0.0, which employs the STAR sequence aligner. The reference genome was the human genome GRCh38 or the mouse genome mm10. Overall, 36,000 cells passed the quality control. To remove the cells with low quality, cells with gene number over 500 and the ratio of mitochondria lower than 0.20 were maintained, and genes with at least one feature count in more than three cells were used for the following analysis. We determined gene expression counts using unique molecular identifiers (UMIs) for each cell barcode-gene combination. Following alignment, we filtered cell barcodes to identify those which contain cells using the approach implemented in Cellranger 3.0.2, and only these barcodes were considered for downstream analysis including clustering and cell type identification, differential expression analysis by Seurat (version 4.0.1).

### Dimensionality reduction

To enable unsupervised clustering and cell type identification, we perform dimensionality reduction with principal component analysis (PCA) on the combined set of samples for each tissue. To visualize the data, we further reduced the dimensionality of all 26,231 cells using Seurat and used t-SNE to project the cells into 2D space. The steps include: 1) Using the LogNormalize method of the “Normalization” function of the Seurat software to calculate the expression value of genes; 2) PCA (Principal component analysis) analysis was performed using the normalized expression value, within all the PCs, the top 10 PCs were used to do clustering and t-SNE analysis; 3) To find clusters, selecting weighted Shared Nearest Neighbor (SNN) graph-based clustering method. Marker genes for each cluster were identified by the “bimod” (Likelihood-ratio test) with default parameters via the FindAllMarkers function in Seurat (4.0.1). Filter Fold Change>1.5 and FDR<0.1 from the calculation results of FindMarker, and then sort to top10 gene as the marker gene.

### V(D)J data processing

### V(D)J assembly and annotation

Cell Ranger for V(D)J (https://support.10xgenomics.com/single-cell-vdj/software/overview/welcome) is a set of analysis pipelines that process Chromium single cell 5′ RNA-seq output to assemble, quantify, and annotate paired V(D)J transcript sequences. Cell Ranger workflow starts by demultiplexing the Illumina sequencer's base call files (BCLs) for each flow cell directory into FASTQ files.

### Cell barcode and UMI correction

Cell Barcode must be on static list of known cell barcode sequences. The barcode may be 1 mismatch away from the list if the mismatch occurs at a low-quality position, it is then corrected. UMIs that are 1 mismatch away from a higher-count UMI are corrected to that UMI if they share a cell barcode.

### Read trimming

Trim known adapter and primer sequences from the 5′ and 3′ ends of reads using the cut adapt tool. This tool uses Smith Waterman alignment and allows for a small number of differences from the expected primer sequences.

### Read filtering

Cell Ranger aligns reads to all the V(D)J gene segments included in the reference. Read-pairs that exceed a specified alignment score and include at least one 15bp exact match against at least one of the reference segments are included in the set of reads to be assembled. These mappings are not in full alignments and are only used for filtering reads before assembly.

### Cell calling

Even though we see many putative cell barcodes in the data, only a fraction of them correspond to droplets that truly contained a cell. The remaining droplets generate background reads. Cell calling algorithm is to select the barcodes corresponding to droplets that contained cells using a 2-component mixture model. Cell calling is performed independently of V(D)J read filtering and assembly.

### Flow cytometry analysis

PBMCs were isolated using Ficoll (GE Healthcare, USA) gradient centrifugation, and were suspended in flow cytometry staining buffer (BD Pharmingen, USA) at a final concentration of 10^7^ cells/ml. After incubation with Fixable Viability Stain, BD Horizon™ Brilliant Stain Buffer, and Fc-blocking antibodies (BD Pharmingen, USA), cells were labeled with antibodies specific for surface markers at 4° C for 30 min. Cells were surface-stained with anti-CD4-FITC, anti-CD3-AF700, anti-CD8-Percp.cy5.5, anti-CD11b-BV510, anti-CD14-BV605, anti-CD16-BV421, anti-CD19-APC, anti-CD45-BV650, anti-CD56-PE (Biolegend, USA). To conduct intracellular cytokine staining, the cells were washed, fixed, permeabilized using the BD Cytofix/Cytoperm™ Fixation/Permeabilization Solution Kit, and then stained using anti-IL-10-APC-R7 antibodies (BD Pharmingen, USA). The results were analyzed using FlowJo software version 10.0.7.

### Statistical analysis

The results were shown as the mean ± standard error of the mean depending on normality. Comparisons between two groups were performed using Student’s t tests (normal distribution). Spearman rank correlation analysis was used to determine the statistical correlation between variables. Statistical significance was accepted at *p* < 0.05. All statistical analyses were performed and images were generated using IBM SPSS Statistics 26 and GraphPad Prism (version 7.0), respectively.

## RESULTS

### Single-cell transcriptional profiling of peripheral immune cells in patients with TAO

To understand the heterogeneity of single-cell transcriptomes at different stages of the disease, we categorized TAO patients into two groups according to the CAS criteria: those with notable orbital inflammation were designated as active group, while those with minimal clinical symptoms were classified as the inactive group [32]. PBMCs were isolated from the whole blood of each subject for further study.

**Figure 1:**
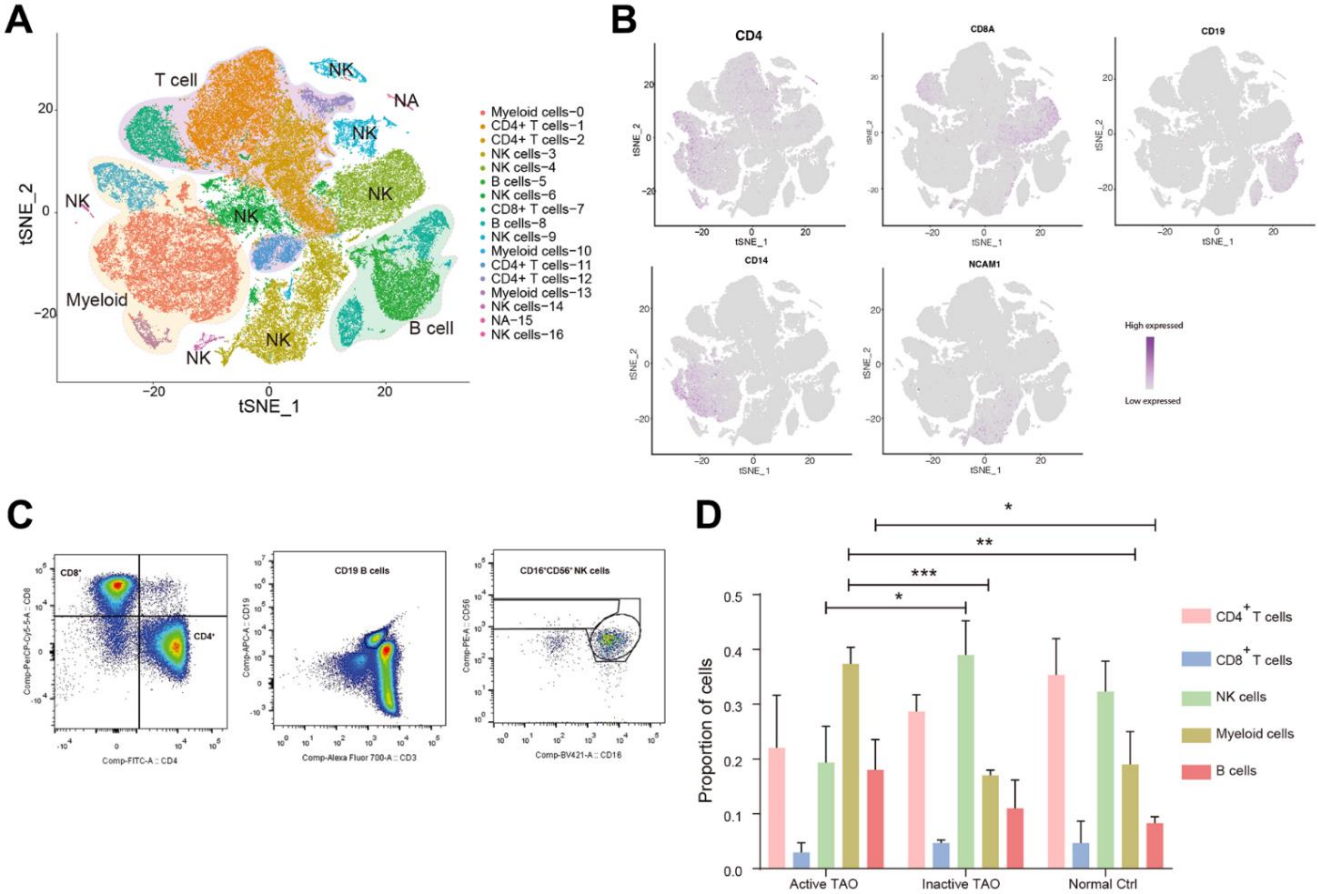
**Single-cell profiling of blood immune microenvironment in TAO patients.** (**A**) A total of 5 main PBMC cell types were identified in PBMC by automated annotation using SingleR software. Color scheme denoting clusters was labeled with inferred cell types. (**B**) The t-SNE feature plots showed expression of marker genes of the five cell types. (**C**) FACS plots validated that CD4^+^ T cells, CD8^+^ T cells, B cells, and NK cells were present in the peripheral blood. Blood sample from one normal control was used for analysis. (**D**) Bar plots showing the percentage of five PBMC cell types from TAO patients and NCs. Error bars show SEM (* *p*<0.05, ** *p*<0.01, *** *p*<0.001).

We performed scRNA-seq on a total of nine samples using the 10x Genomics Chromium platform (Supplementary Figure 1A). After initial quality control, a total of 93,007 PBMCs were obtained for subsequent analysis, comprising 23,051 cells from active TAO, 34797 cells from inactive TAO, and 35,159 cells from normal controls (NCs) (Supplementary Table 1). Graph-based clustering analysis identified 17 distinct clusters. Following manual confirmation of the cell type annotations, we successfully categorized the clusters into five major PBMC cell types. These included various populations of CD4^+^ T cell (*CD4^+^*), NK (*CD56^+^*), CD8^+^ T cells (*CD8A^+^*), B cells (*CD19^+^*), and myeloid (*CD11b^+^*) (Figure 1A, 1B and Supplementary Figure 1B and Supplementary Table 2). The presence of these five cell types identified by scRNA-seq was validated by flow cytometry analysis (Figure 1C). Notably, 12 of the 17 cell clusters (70.6%) contained at least 100 cells from multiple individual samples, with a median of 9 individual samples represented within each cluster (Supplementary Figure 1C).

We next sought to examine the variations in the prevalence of these populations according to the disease stage. We calculated the percentage of each cluster identified through scRNA analysis relative to the total number of clusters for each patient sample (Figure 1D and Supplementary Table 3). Our findings revealed a remarkable diversity in the immune profiles across patients, with active TAO showing significant infiltration of B cells (*p*=0.047) and myeloid cells infiltration (*p*=0.008). In contrast, NK cells were predominantly observed in inactive TAO (*p*=0.022) and NCs (*p*=0.059). Although the difference did not reach statistical significance, both inactive TAO and NCs samples showed an enrichment of CD4^+^ T cells. The proportion of CD8^+^ T cells exhibited minimal changes across there sample groups. These findings emphasized the alterations in PBMC composition in individuals with active TAO, underscoring the importance of gaining a deeper understanding of the diversity of cell subtypes and states that related to disease phases.

### Regulatory B cells involve in immune regulation during TAO

**Figure 2:**
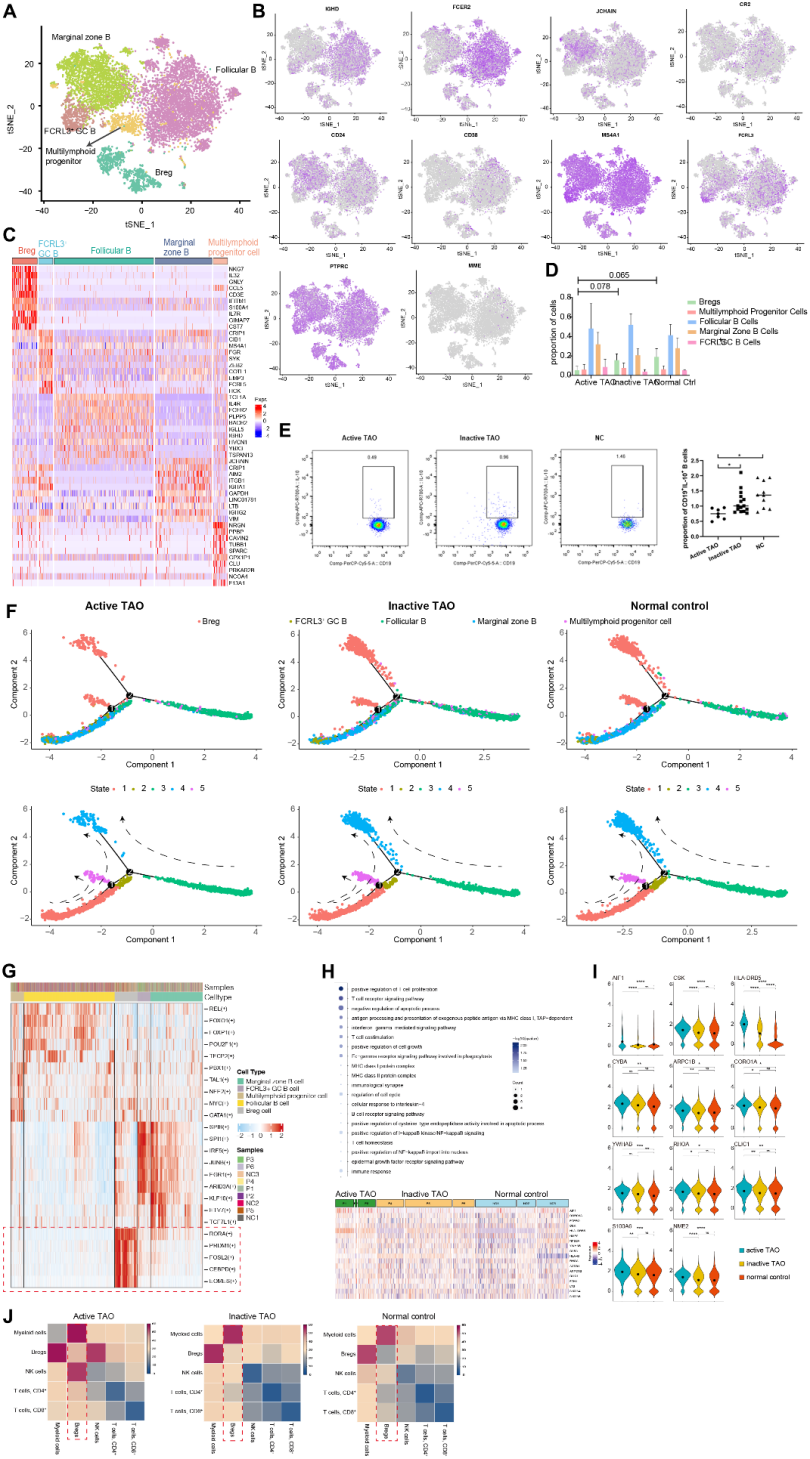
**scRNA-seq revealed Bregs with immunoregulatory properties in TAO.** (**A**) t-SNE plot showing the annotated B cells captured from Figure 1B. Each dot denotes an individual B cell, color denotes cluster origin. (**B**) The t-SNE feature plots showed expression of marker genes within individually identified B cell populations. (**C**) Heatmap of DEGs of each B cell subpopulation provides phenotypic information for individual B cell populations. Expression values are scaled between minimum and maximum expression for each gene across all clusters. (**D**) Prevalence of each B cell subpopulation within all samples from TAO patients with different activity and normal controls. (**E**) Representative FACS dot plots showed the proportion of Bregs from a patient with active TAO, a patient with inactive TAO, and a normal control (left). Scatter plot showing the frequency of Bregs in active TAO, inactive TAO, and NC group (right). (**F**) Trajectory analysis of B cell subsets in NCs and patients with TAO. Upper: distribution of the B cell subpopulations on each of the branches. Lower: the relative proportion of B cell subpopulations in each state. (**G**) Significantly activated motifs in the differentiation process colored by cell clusters. (**H**) Heatmap showing upregulated DEGs in active TAO patients’ Bregs. The upregulated GO terms enriched in the DEGs are listed with *p*-value and gene numbers. (**I**) Violin plots showing upregulated DEGs of Bregs. The diamond inside the violin plot represents the mean. (**J**) Heatmap showing the distribution of interaction pairs across the cell types.

To comprehensively understand the heterogeneity of B cells and their role in TAO, we conducted further analysis to identify their distinct subpopulations based on RNA-seq data. Using graph-based clustering, we identified five subpopulations comprising a total of 9493 B cells (Figure 2A). Furthermore, we thoroughly assessed the expression of a comprehensive panel of genes associated with B cell lineage to gain deeper insights into their distinct features. The follicular B cells were characterized by the expression of *IGHD* (IgD) and *CD23* (also known as FCER2). The marginal zone B population exhibited high expression of *JCHAIN* (IgM) and *CD21* [33]. Bregs were identified based on the expression of *CD1d, CD5, CD19, CD24, CD38,* and *CD27* [14]. Additionally, germinal center B cells were recognized as a cluster displaying high levels of *CD20* (MS4A1), *CD38*, and *FCRL3* [34]. Lastly, a small number of B cells were identified as multilymphoid progenitor cells, expressing *CD45RA* (PTPRC), *CD34*, and *CD10* (MME) (Figure 2B). The top ten different expression genes (DEGs) for each B cell subpopulation provided additional evidence for our annotation and supported the distinct identity and functional characteristics of each B cell subcluster (Figure 2C).

We subsequently examined the B cell composition in each sample during different phases of the disease. All B cell clusters were observed in each PBMC sample (Supplementary Figure 2A). Although there was no significant difference between the relative proportions of Bregs for the three groups, the proportions of Bregs were lower in active TAO when compared to inactive TAO and normal control groups (*p*=0.078 and *p*=0.065, respectively) (Figure 2D). Despite the observed differences not reaching statistical significance, this result indicated a potential trend in the data. To validate these findings, we conducted flow cytometry analysis and used CD19^+^IL-10^+^ as the marker for identifying Bregs cell population. IL-10 had been widely recognized as the hallmark of Breg due to its ability to suppress pro-inflammatory responses [35]. This analysis confirmed that Bregs from active TAO exhibited a reduced frequency compared to those in inactive TAO and the NC group (Figure 2E).

Trajectory analysis was performed to elucidate the dynamic relationships among different B cell subgroups. The analysis uncovered 3 branchpoints and 5 distinct states. State 1 was characterized by a simultaneous distribution of marginal zone B cells and FCRL3 high GC B cells. State 2 consisted of a small fraction of follicular B cells. State 3 predominantly comprised follicular B cells. State 4 and state 5 were primarily composed of Bregs. Additionally, all states exhibited diffused multilymphoid progenitor cells (Figure 2F and Supplementary Figure 2B). In line with previous findings, we observed a reduced cell density of Bregs in state 4 and state 5 in active TAO. According to the SCENIC analysis, a distinct set of genes that regulated by different transcription factors was found to be specifically expressed in Bregs while being almost absent in other cell types (Figure 2G). In Bregs, the genes regulated by *RORA*, *CEBPD*, *PRDM1*, *FOSL2*, and *EOMES* displayed a notably higher expression level. *RORA*, a crucial regulator in cell differentiation and immunity [36], had been recently implicated in B cell development [37]. *CEBPD*, known to modulate various biological processes, including cell differentiation, proliferation, and cell death, played a pivotal role in regulating immune and inflammatory responses [38]. The DNA-binding protein Blimp-1, encoded by PRDM1, dynamically influences the generation, differentiation, and IL-10 production of Bregs [39]. *FOSL2* (also known as *FRA2*) acts as a key enhancer in regulating early B cell proliferation and differentiation [40]. While *EOMES* had been recognized as essential for the differentiation of T cells and NK cells [41, 42], its role in B cell development remains to be elucidated.

The GO enrichment analysis revealed that Bregs exhibited upregulated genes associated with immunoregulation (*AIF1, CSK, HLA-DRB5, CYBA*), cell cycle progression, signal transduction, and apoptosis (*ARPC1B, CORO1A, YWHAB, RHOA, CLIC1, S100A6, NME2*) (Figure 2H, 2I). Notably, in active TAO, the upregulated genes of Bregs were enriched in inflammation-related GO terms and cellular process-related GO terms. These findings strongly suggested that Bregs may play a crucial role in modulating inflammation and immune responses during the TAO process. According to the CellPhoneDB analysis, Bregs exhibited a high frequency of communication with myeloid cells (Figure 2J). This finding indicates that Bregs and myeloid cells are likely to engage in coordinated immunoregulatory processes during TAO.

### scBCR-seq analysis uncovered a notable increase in the diversity of BCRs in active TAO

**Figure 3:**
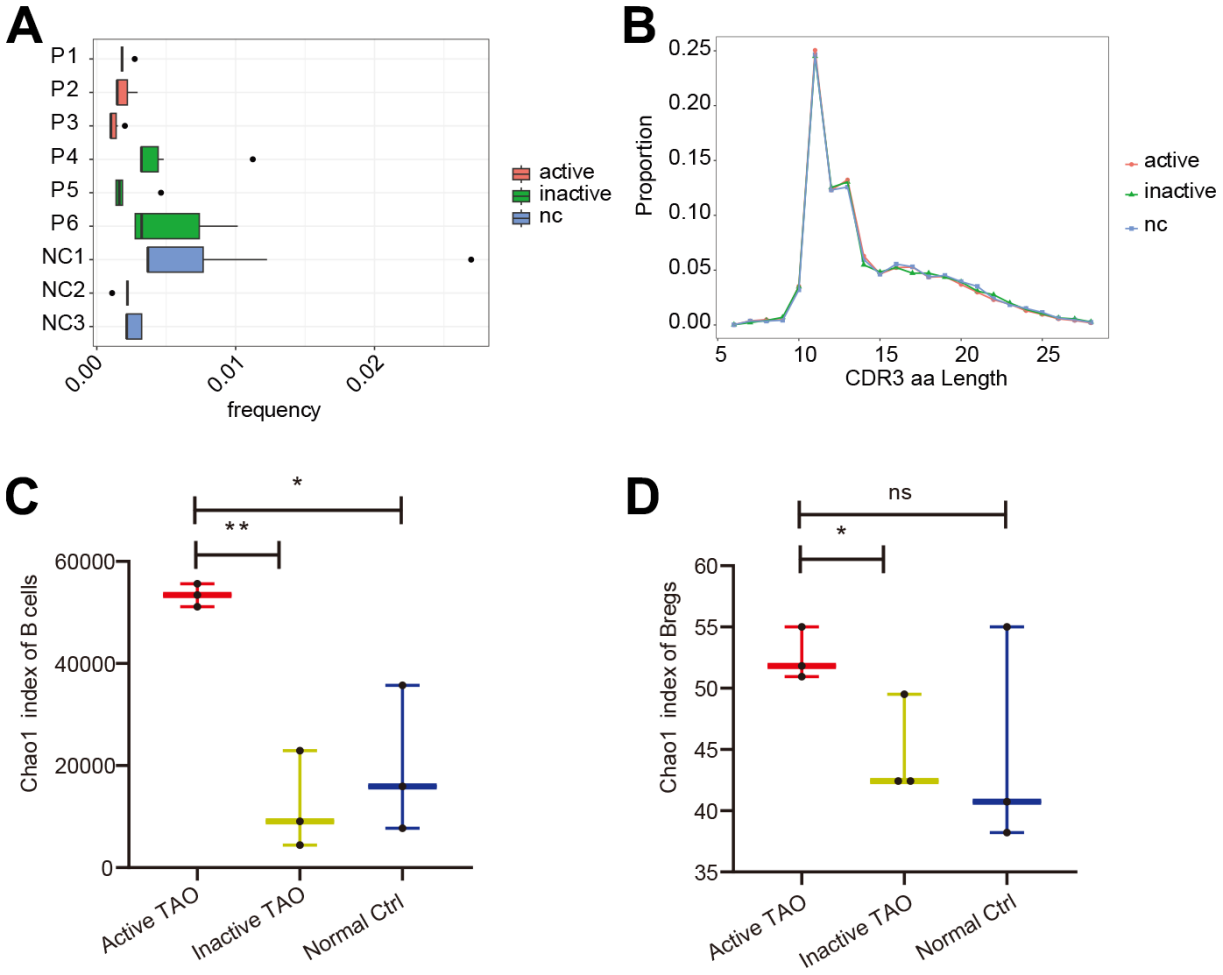
**scBCR-seq revealed increased diversity of BCRs in active TAO.** (**A**) The box plot showed the top 10 high-frequency B-cell clonotypes for each sample in active TAO, inactive TAO, and NC group. (**B**) The distribution of the length of CDR3 amino acid sequence in active TAO, inactive TAO, and NC group. (**C**) Box plots of Chao1 index for each sample were used to compare the diversity of CDR3of B cells (* *p*<0.05, ** *p*<0.01). (**D**) Box plots of Chao1 index for each sample was used to compare the diversity of CDR3 of Bregs (* *p*<0.05, ** *p*<0.01).

To allow for a comprehensive analysis of both gene expression and BCR diversity in the B cell populations, scBCR-seq was performed for the PBMC samples used in scRNA-seq analysis. In the active TAO, the proportion of the top 10 clonotypes (0.17%) exhibited a significant decrease compared to in the inactive group (0.37%, *p*=0.010) and the NC group (0.41%, *p*=9.31E-05) (Figure 3A). However, analysis of the length of the complementarity determining region 3 (CDR3) did not show any noticeable changes (Figure 3B). When examining the diversity of CDR3, as quantified by the Chao1 index, a significant increase was observed in the active TAO group compared to both the NC group (*p*=0.016) and the inactive TAO group (*p*=0.002) (Figure 3C). A similar trend was noted in Breg cells (Figure 3D), with a significant increase compared to the NC group (*p*=0.011) but no statistically significant difference between the active TAO group and the NC group (*p*=0.213).

These findings suggested that while the frequency of the top 10 clonotypes was reduced in active TAO, there was an increased diversity of CDR3 sequences in this condition, which might have implications for the regulation of immune responses and contribute to the pathogenesis of active TAO.

### Alteration in configuration of myeloid cells in active TAO

**Figure 4:**
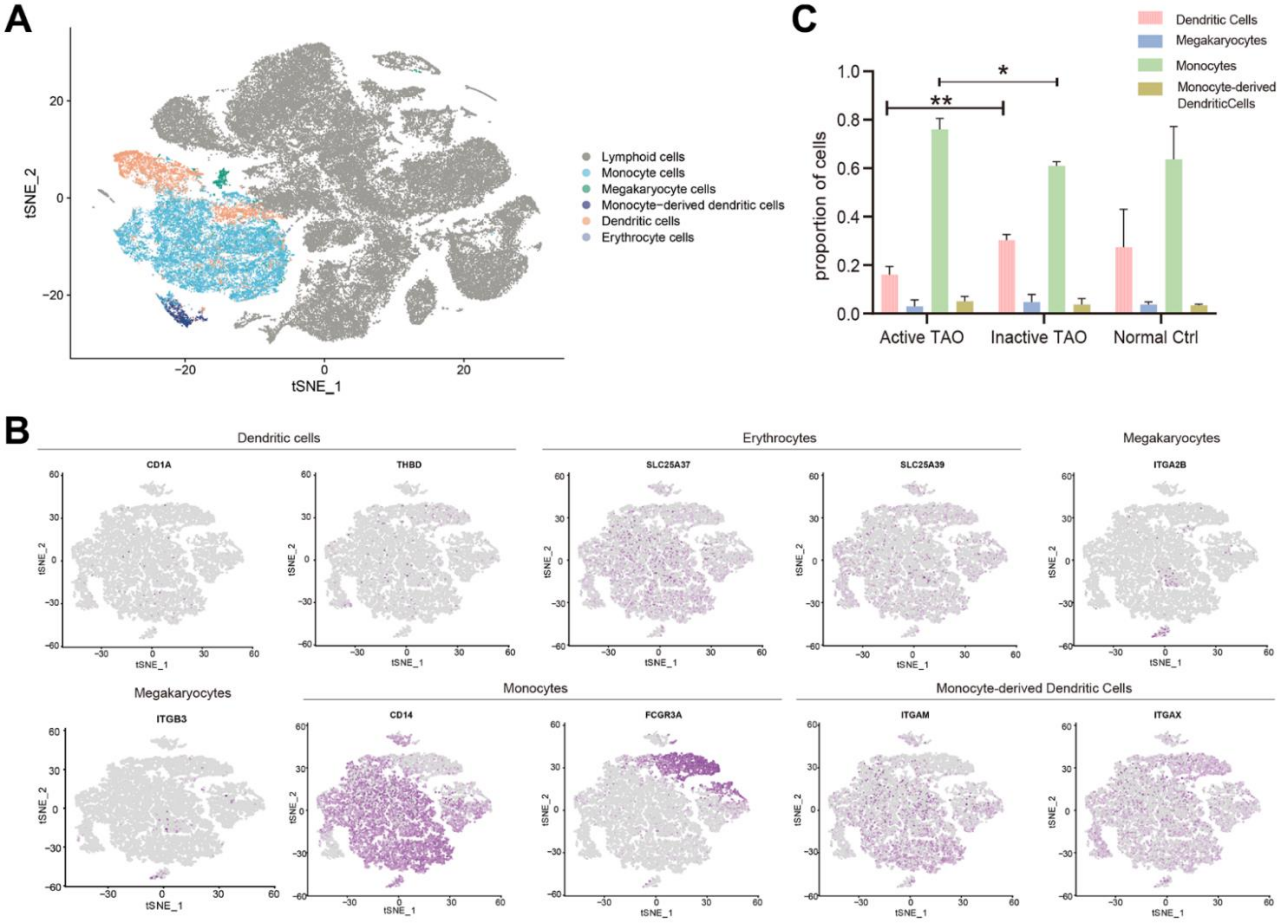
**Analysis of the myeloid landscape reveals decreased DCs and increased monocytes in active TAO.** (**A**) Sub-clustering of myeloid cells identifies 5 cellular categories, including monocytes, megakaryocytes, monocyte-derived dendritic cells, dendritic cells, and erythrocytes. (**B**) tSNE feature plot representation of marker genes expression within individually identified myeloid cells. (**C**) Bar plots showing the percentage of myeloid subclusters in active TAO, inactive TAO, and NC group (* *p*<0.05, ** *p*<0.01).

To assess the involvement of myeloid cells in TAO, we identified the major subtypes of myeloid cells and examined their configuration in active TAO. The analysis revealed five distinct myeloid cell populations, namely monocytes, megakaryocytes, dendritic cells (DCs), monocyte-derived DCs, and erythrocytes (Figure 4A and Supplementary Figure 3A). The identification of these cell types was based on differential gene expression and examination of established lineage markers (Figure 4B and Supplementary Figure 3B). A comparison between active TAO and inactive TAO unveiled notable differences in the proportions of monocytes and DCs. Active TAO patients displayed a significantly higher proportion of monocytes (*p*=0.006), while the proportion of DCs in active TAO was significantly lower compared to the inactive TAO group (*p*=0.003) (Figure 4C). These findings suggested that increased levels of monocytes and decreased populations of DCs might be characteristic of the active disease state in TAO patients. To further understand the clinical relevance of quantitative changes of Bregs, DCs, and monocytes in TAO, we examined correlations between the proportion of these cells and TAO disease activity and severity. As shown in Supplementary Figure 4, the proportion of monocytes were positively correlated with the CAS score of TAO patients (*p*=0.0004, r^2^=0.9668) (r^2^ represented the coefficient of determination in Spearman rank correlation analysis). And the proportion of Bregs were negatively correlated with the NOSPECS score of TAO patients (*p*=0.0158, r^2^=0.8021). However, no significant correlations were found between the proportion of Bregs, DCs and the CAS score of TAO. Similarly, the proportion of DCs and monocytes showed no correlations with NOSPECS score of TAO (Supplementary Table 4 and Supplementary Figure 4).

### DCs might involve in the inflammatory process during active TAO

**Figure 5:**
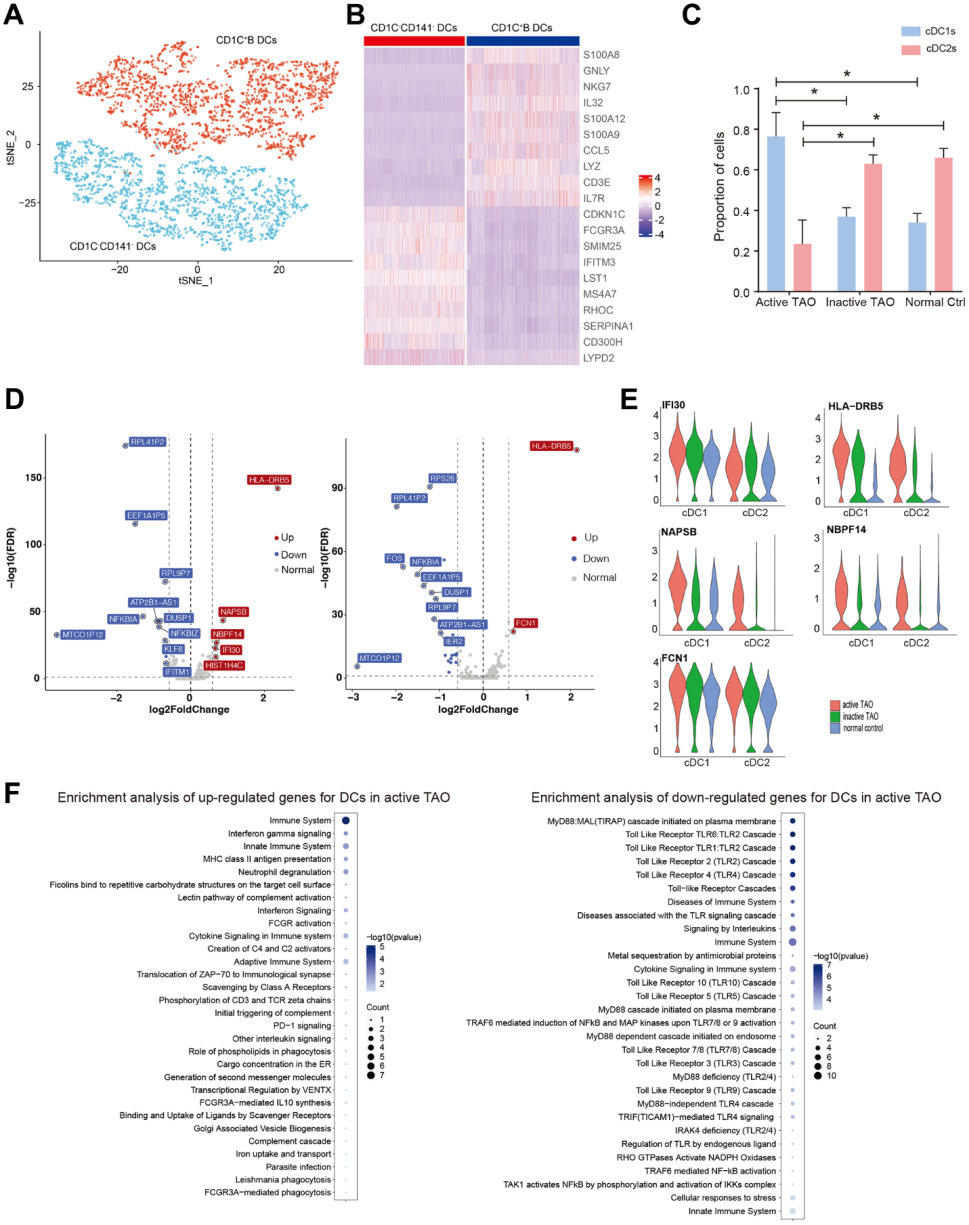
**Activation of DCs may be associated with the inflammation during active TAO.** (**A**) tSNE plot of subgroups of DCs. (**B**) Heatmap of DEGs between three different DC subgroups. (**C**) Bar chart showing the relative proportion of CD1C^-^CD141^-^ DCs and CD1C^+^B DCs from active TAO, inactive TAO and NCs (* *p*<0.05). (**D**) Volcano plots showing DEGs of CD1C^-^CD141^-^ DCs (left), and CD1C^+^B DCs (right) from active TAO patients and NCs. (**E**) Violin plots showing the expression of *HLA-DRB5*, *IFI30*, *NAPSB*, *FCN1*, *MT-ATP6*, *FTL*, *SERPINA1*, *LTA4H*, and *NBPF14* in CD1C^-^CD141^-^ DCs and CD1C^+^B DCs from active TAO patients and NCs. (**F**) Representative Reactome terms enriched by upregulated (left) and downregulated (right) DEGs of DCs in active TAO.

DCs were subdivided into three distinct subclusters. Cluster 0 was designated as cDC1s, which was characterized by high expression of *CDKN1C*, *FCGR3A*, *SMIM25*, *IFITM3*, *LST1*, and *MS4A7*, and low expression of *CD1C* and *CD141*. Clusters 1 and 2 were identified as cDC2s, which exhibited elevated expression levels of inflammatory genes such as *CD14*, *S100A8*, *GNLY*, *NKG7*, *IL32*, and *S100A12* (Figure 5A, 5B). The proportion of cDC1s was significantly higher in active TAO, whereas cDC2s were more prevalent in inactive TAO and NCs (Figure 5C). Given that cDC1s and cDC2s perform distinct roles in immune regulation – with cDC1 being specialized in inducing Th2 response and cDC2 subclusters exhibiting a more potent ability to induce Th1 response [42], we further examined their gene expression profiles. Regarding cDC1s, the genes associated with MHC II antigen presentation (*HLA-DRB5*, *IFI30*), immune activation (*NAPSB*), and developmental and neurogenetic diseases (*NBPF14*) were highly expressed in active TAO compared to NCs (Figure 5D, 5E). For cDC2s, higher levels of *HLA-DRB5* and *FCN1* were observed in active TAO compared to NCs (Figure 5D, 5E). Moreover, for both cDC1 and cDC2, most of the related genes, including *HLA-DRB5*, *NAPSB*, *NBPF14*, *IFI30*, and *FCN1*, also exhibited relatively higher expression levels in active TAO compared to inactive TAO (Supplementary Figure 5A and Figure 5E). Enrichment analysis revealed that for both DCs the upregulated genes in active TAO were primarily associated with various processes related to inflammation, immune activation pathways, and complement activation pathways (Figure 5F). Interestingly, the downregulated genes were predominantly associated with Toll-like receptor (TLR) cascade pathways which are essential for DCs activation and maturation [43, 44]. These results suggested that the activation of DCs might be impaired in active TAO, and the observed inflammation during this active phase could be closely related to these cells.

### Monocytes play a role in active TAO by regulating inflammatory cytokine production

**Figure 6:**
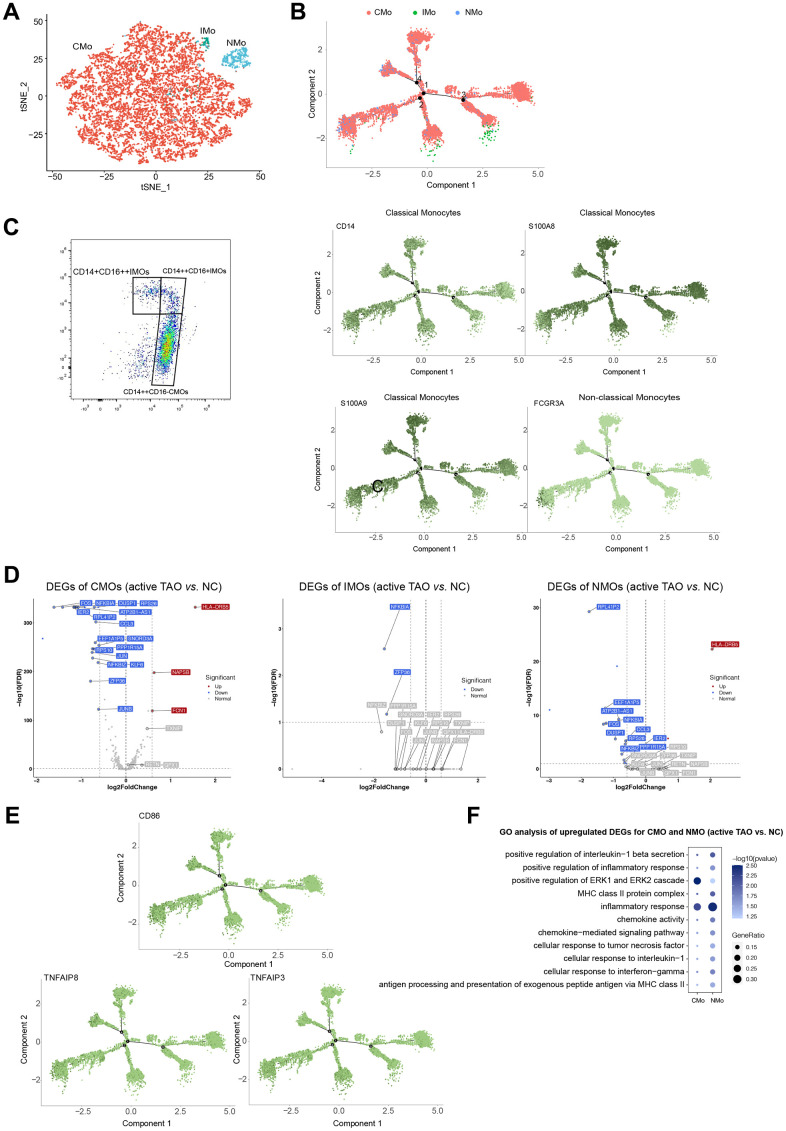
**Monocytes displayed increasing inflammatory cytokine production and cos-stimulatory molecule expression in active TAO.** (**A**) tSNE plot showing clusters of monocyte subsets. (**B**) Pseudotime analysis of monocytes recapitulate known lineage relationships, with CMOs (*CD14*^+^*S100A8*^+^ *S100A9*^+^) branching into either NMOs (*FCGR3A*^+^) or IMOs (*CD14*^++^ *FCGR3A*^+^). (**C**) FACS plots validated that CMOs, IMOs, and NMOs were present in the peripheral blood. Blood sample from one normal control was used for analysis. (**D**) Volcano plots showing DEGs of CMOs, IMOs and NMOs in active TAO/NC comparison. (**E**) Inflammatory cytokines and costimulatory molecules (CD86 and TNF) increased in CMOs and NMOs in active TAO. (**F**) Representative GO terms enriched by upregulated DEGs of monocytes in active TAO.

From a total of 12,959 monocytes, our clustering analysis successfully identified three distinct subpopulations. The first cluster was characterized as classical monocytes (CMOs), which exhibited high expression levels of *CD14*, *S100A8*, and *S100A9*. The second cluster was identified as non-classical monocytes (NMOs), characterized by their high expression of *FCGR3A* (also known as *CD16*). The third cluster was recognized as intermediate monocytes (IMOs), with both *CD14* and *FCGR3A* highly expressed (Figure 6A, 6B and Supplementary Figure 5B). To validate these monocyte subclusters, flow cytometry analysis was conducted, confirming CMOs as CD14^++^CD16^-^, NMOs as CD14^+^CD16^++^, and IMOs as CD14^++^CD16^+^ (Figure 6C).

Further comparisons were made to assess the proportions of the three monocyte subsets among different groups. Although not statistically significant, it was observed that NMOs and IMOs were more abundant in inactive TAO when compared to active TAO (Supplementary Figure 5C, 5D). Trajectory inference analysis revealed that monocytes followed a developmental trajectory starting from CMOs and branching towards NMOs and IMOs states (Figure 6B). These observations of developmental state alterations in monocyte cells during TAO suggest their potential roles in the disease.

The results of the DEGs analysis revealed that CMOs exhibited significantly higher expression of genes such as *HLA*-*DRB5*, *NAPSB*, and *FCN1* in active TAO compared to other groups. Similarly, NMOs in active TAO showed relatively higher expression of genes such as *HLA-DRB5* and *SNORP3A* (Figure 6D and Supplementary Figure 5E). Both CMOs and NMOs exhibited higher expression of *TNF* and *CD86* in active TAO, but these genes were significantly lower in expression in IMOs compared to the normal control group (Figure 6E). Enrichment analysis of DEGs suggested that inflammation-associated pathways and MHC II antigen presentation were upregulated in CMOs and NMOs (Figure 6F). These data indicated that monocytes play a role in regulating pro-inflammatory effects during active TAO.

### Crosstalk among Bregs and myeloid cells in TAO

**Figure 7:**
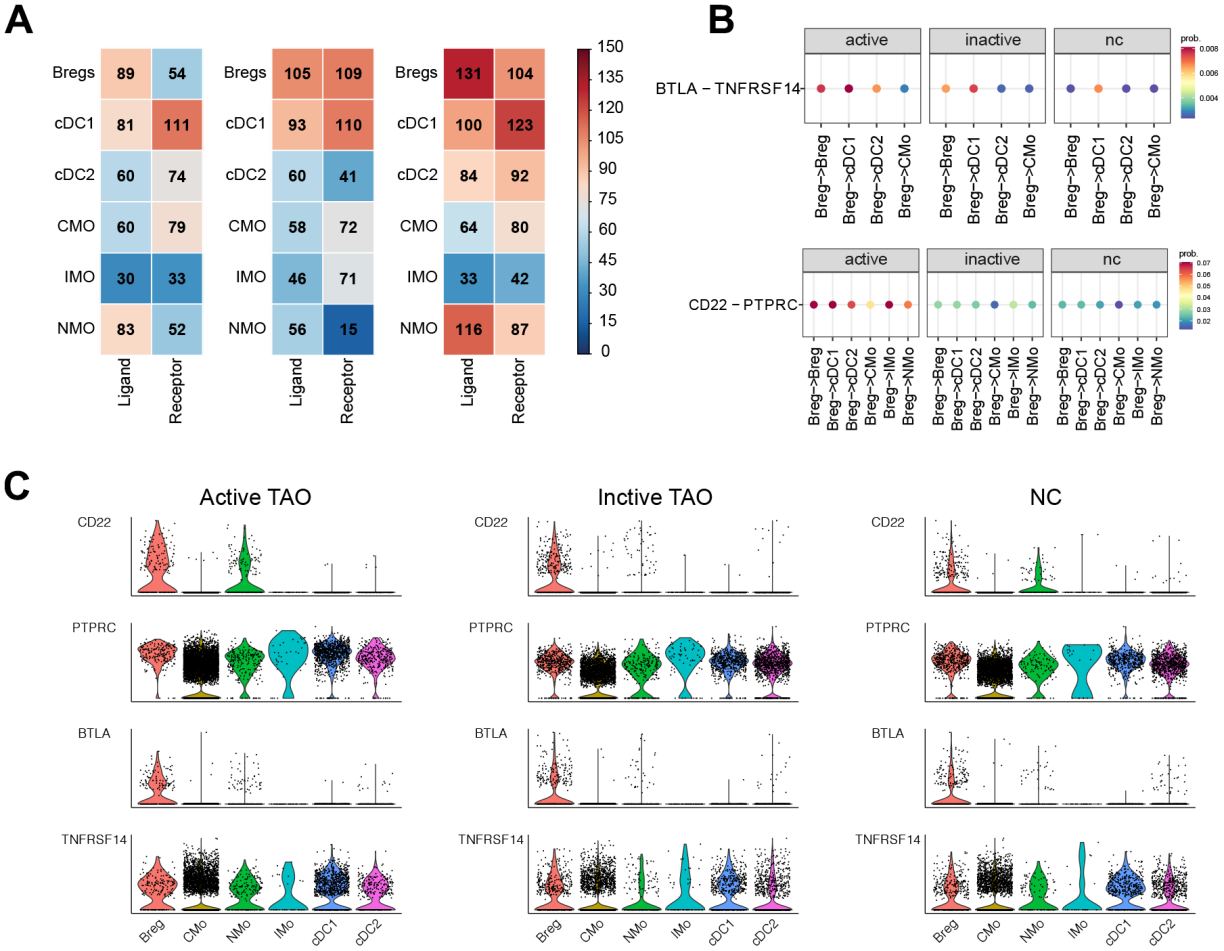
**CellChat analysis based on scRNA-seq showed the crosstalk between Bregs and myeloid cells in the immune network.** (**A**) Heatmap showed the distribution of interaction pairs across the cell types. (**B**) Chord plots showed the interactions of ligand/receptor pairs between active TAO, inactive TAO, and NC group. (**C**) Violin plots showed the expression levels of LTA and TNF family cytokines in each immune cell type for both TAO and HC groups.

Cell interactions among Bregs, DCs, and monocytes in the context of active TAO were further examined. This analysis identified a total of 1,352 pairs of significant interactions. Remarkably, Bregs were found to exhibit the highest number of ligands, whereas cDC1 subclusters showed the highest number of receptors. Notably, Bregs displayed an increased number of ligands in active TAO when compared to both inactive TAO and the normal control group (NCs) (Figure 7A).

We conducted a detailed analysis of the expression patterns of corresponding receptors and ligands associated with inflammation and immune activation. Compared to inactive TAO, and normal control (NC) groups, it was observed that the *CD22-PTPRC* interaction between Bregs and cDC1s, cDC2s, IMOs, NMOs, or CMOs were significantly stronger in active TAO patients (Figure 7B). Moreover, we observed robust inhibitory interactions in active TAO, such as BTLA-TNFRSF14 between Bregs and cDC1s, cDC2s, or IMOs. This finding suggests a potentially more immunosuppressive role for Bregs in the context of active TAO. (Figure 7B).

Through examination of the expression levels of genes having potential role in immunosuppressive and inflammatory processes, such as *CD22*, *PTPRC*, *BTLA*, and *TNFRSF14* in these cell subclusters, we found that *CD22* expression was increased in Bregs during active TAO. Furthermore, the expression levels of *BTLA* and *TNFRSF14* showed an overall increase in Bregs, DCs, and monocytes in active TAO when compared to inactive TAO and the normal control (NC) group (Figure 7C).

These findings suggest intricate regulatory interactions between Bregs, DCs, and monocytes during active TAO, which may contribute to the inflammatory and immune responses observed in the disease.

We know that due to limited understanding of the potential immune aberrations of TAO, it is difficult to develop new and effective therapies and interventions. In the above study, the immune components in peripheral blood of TAO patients and healthy subjects were analyzed by single-cell RNA sequencing and B-cell receptor profile (BCR), and it was found that the proportion of regulatory B cells (Bregs) and type 2 conventional dendritic cells (dc) decreased significantly during the active phase of TAO patients. In contrast, the proportion of type 1 dc is significantly increased, and the transition of B cells to the Breg phenotype may be impaired. In other words, the inflammatory process of TAO is related to the dysfunction of immune regulation. At the same time, abnormalities in the peripheral immune system are primarily driven by enhanced interactions between Breg, dc, and monocytes. There is a link between these identified immune aberrations and potential therapeutic targets, and these findings could provide new therapeutic approaches or interventions for TAO.

These results indicate that some immune cell populations change during TAO activity. The expression level of inflammatory genes is increased, and different inflammatory genes play different roles in immune regulation, that is to say, the change of immune cell population plays a positive role in immune regulation. In addition, there are complex regulatory interactions between different cells during TAO activity, which may contribute to the inflammatory and immune responses observed in disease. In addition, dynamic changes in the activity of key regulators suggest that TAO activation appears to involve inflammation and immune dysfunction. Overall, our findings provide comprehensive insights into the molecular regulation and cell remodeling of the active phase of TAO at the single-cell level, with positive clinical implications for suggesting the potential role of monocytes in disease through the observed developmental status of monocytes during TAO.

## DISCUSSION

A better understanding of immune cell involvement in TAO is crucial for unraveling the pathogenesis of this condition and for identifying effective treatment approaches. In our study, we undertook a comprehensive exploration of the single-cell transcriptome and the immune phenotypes of B cell receptors in individuals with active TAO, inactive TAO, and NCs. Through our analysis, we identified five major cell types and emphasized the distinct transcriptional heterogeneity within B cell and myeloid cell subsets. These findings provided valuable insights into the complex immune processes underlying TAO and may pave the way for further research and the development of targeted therapeutic interventions. Furthermore, we observed a potential defect in the shift of B cells towards the Breg phenotype during active TAO, which might be associated with the modulation of inflammation and immune responses. Bregs are known to exert their suppressive effect on immune responses primarily through the secretion of anti-inflammatory cytokines such as IL-10, TGF-β, and IL-35, as well as the expression of other suppressive molecules like PD-L1, granzyme B, CD39, CD73, and the aryl hydrocarbon receptor (AhR) [14]. However, it remains unclear whether and when Bregs produce these anti-inflammatory cytokines during the disease progression, and the cell fate determination in the context of TAO remains to be elucidated. Further research is needed to study the dynamic transition of Bregs in TAO and their potential as therapeutic targets for modulating the inflammatory and immune processes involved in this disease.

The differential gene expression analysis supported the inflammation regulatory capacity of Bregs in active TAO. In active TAO, Bregs expressed high levels of *AIF1, CSK,* and *CYBA*. * AIF1* could activate the expression of *IRF8* (the main transcription factor for the generation of cDC1) and interact with protein kinase C to drive NFκB signaling cascades [45]. Inhibiting AIF1 has been proved to restrain CD4^+^ T cell effector responses and induce CD25^+^Foxp3^+^ Tregs [46]. CSK, which acts as a negative regulator of Src family of kinases that play critical roles in inflammation [47]. CYBA encoded p22phox, an important component of NADPH-oxidase complex, which was critical for ROS generation [48]. ROS had been proved to be associated with the release of proinflammatory cytokines, hyaluronan synthesis, and proliferation and differentiation of OFs in TAO [49]. Taken together, Bregs might be essential in modulating the inflammation in peripheral blood of TAO, and the dysregulated genes in Bregs during active TAO may be considered as potential blood diagnostic biomarkers.

The proportion and function of Bregs in peripheral blood were observed to vary depending on the type and stage of the disease. In individuals with Hashimoto's thyroiditis, there was a significant decrease in the level of CD19^+^CD24^hi^CD38^hi^ B cell subsets (B cell populations known for producing IL-10) compared to healthy controls [50]. Similarly, patients with systemic lupus erythematosus [51] and rheumatoid arthritis [52], exhibited reduced counts of IL-10-producing B cells, which resulted in an inability to suppress the secretion of pro-inflammatory cytokine by T cells. This pattern was also observed in patients with TAO [53] and newly diagnosed Graves’ Disease [54]. A recent study further demonstrated that Bregs from active TAO patients were defective in suppressing the activation of IFN-γ^+^ and IL-17^+^ T cells *in vitro* [55]. In our study, we noted that the frequencies of IL-10-producing Bregs in active TAO were slightly lower than those in inactive TAO patients and healthy controls, consistent with the findings from the studies by Ding and Zha. Further effort is needed to elucidate the potential molecular mechanism underlying the impaired regulatory functions of Bregs in active TAO.

The activation of DCs and the involvement of monocytes in the cascade of autoimmune inflammation were significant contributors to the pathogenesis of active TAO. Percentage of myeloid DCs in PBMC fraction was significantly lower in patients with both active and inactive TAO, compared to patients with GD without TAO and controls [56]. The proportion of CMOs in active TAO was found to be significantly lower than inactive TAO, while the proportion of IMOs was significantly higher in active TAO [57]. These observations shed light on the immune imbalance that occurs during TAO development, providing valuable insights for further research and potential therapeutic strategies. It was worth noting that varying amounts of circulating myeloid DCs and monocytes had been observed in TAO [56] and in several other autoimmune diseases and inflammatory disorders [58]. In our study, we made a notable discovery that DCs were significantly decreased in active TAO [56]. Additionally, we found that cDC2 subclusters were less enriched in active TAO. Both DCs and monocytes displayed upregulated genes related to inflammation, immune activation, and costimulatory molecules in active TAO.

Through cell interaction analysis, we observed that Bregs might modulate DC functions and consequently influence immune responses which possibly achieved by controlling the number of DCs through Fas-FasL–mediated apoptosis [59]. In our study, we observed significantly stronger *CD22* signals between Bregs, DCs, and monocytes in active TAO patients. B cells acquired regulatory capacity after being triggered by various factors such as TLR ligands, different pro-inflammatory cytokines, or co-stimulatory molecules [14]. TLR signaling induces the expansion of Bregs and the production of inhibitory molecules, including IL-10, which helps restrict the extent of inflammation [35]. CD22, a major inhibitory receptor on B cells, negatively regulates B cell activation induced by TLR ligands, thereby inhibiting the expansion of Bregs [60]. This suggested that Bregs in active TAO might receive more activation signals from DCs through the CD22-PTPRC axis, leading to a decrease in Bregs and a potential defect in the inhibition of inflammation. Furthermore, another factor contributing to the reduction of Bregs in active TAO patients could be the increased BTLA-TNFRSF14 signals from DCs and monocytes. BTLA (B and T lymphocyte attenuator) was another important co-inhibitory receptor that binds to TNFRSF14 [61]. BTLA is also expressed on innate immune cells, such as DCs and monocytes [61]. Upon ligation with TNFRSF14, BTLA inhibits the proliferation, cytokine production, and upregulation of co-stimulatory molecules in CpG-mediated B cell functions [62]. However, the functions of these signal pathways in the pathogenic mechanism of TAO need further experimental validation. Nevertheless, these observations provided valuable insights into the intricate interactions between Bregs, DCs, and monocytes in the context of TAO and may open potential avenues for future research to better understand the immune dysregulation in this condition.

The diversity of BCR plays a crucial role in determining the autoimmune response [63]. CDR3 is a part of the BCR amino acid sequence responsible for binding to antigens. The diversity of CDR3 amino acid sequences is determined by the V(D)J recombination of variable (V) and joining (J) gene segments in the light chain (kappa and lambda), as well as variable (V), diversity (D), and joining (J) gene segments in the heavy chain, constituting the diversity of CDR3 sequences [64]. In our single-cell BCR-sequencing analysis, we found a significant increase in the diversity of CDR3 amino acid sequences in active TAO. This finding aligns with our hypothesis that inflammation during active TAO may lead to an increase in the diversity of CDR3 sequences. The increased diversity of CDR3 may reflect the broadening of the BCR repertoire in response to various antigens and may be indicative of an ongoing adaptive immune response in active TAO. Understanding the dynamics of the BCR repertoire and its correlation with disease activity could potentially lead to novel insights into the pathogenesis of TAO and provide valuable information for developing personalized therapeutic strategies.

In this study, we depicted a comprehensive peripheral blood cell landscape of TAO, which elaborated the composition and functional changes of peripheral cellular components in TAO. These data enriched our understanding of the pathogenic mechanisms of characteristic changes and provided a database for identifying a sensitive and specific biomarker for TAO.

In addition, various techniques were used in this paper, such as scRNA-seq and flow cytometry, which made the experimental results more realistic and reliable. But in the actual experimental operation process, these techniques have certain limitations. For example, in the process of flow cytometry analysis, the experiment has certain requirements on the state of cells, and different cell states may lead to different experimental results. It is not appropriate to use samples that have been cryopreserved for this experiment, as these conditions may affect the physical state and fluorescence properties of the cells. During the experiment, the cells to be tested need to maintain high activity, low activity cells may lead to non-specific fluorescence staining, affecting the accuracy of the results. In addition, the cell concentration needs to be adjusted before the experiment, and too low the concentration will directly affect the accuracy and reliability of the detection results. Similarly, although single-cell RNA sequencing can obtain gene expression at the cellular level, sample preparation and library building are more expensive, and analysis is more complex and difficult to interpret. Therefore, when applying the above techniques, it is necessary to consider the limitations of the experiment to carry out further experimental verification of the experimental results.

### Data limitations and perspectives

The present study contains a small sample size and lack of functionally validated study of immunosuppressive function of Bregs in the evolution of TAO. However, our study highlighted significant changes in the proportion and transcriptional heterogeneity of B cells, DCs, and monocytes in individuals with active TAO, inactive TAO, and NCs. These findings provided valuable insights into the alterations in immune cell infiltration within the peripheral immune environment during active TAO. Notably, we identified potential inhibitory effects of two ligand-receptor pairs, CD22-PTPRC and BTLA-TNFRSF14, which may contribute to the decrease of Bregs and impaired inhibition of inflammation in active TAO. These potential pathogenic cells and molecules may account for the inflammatory changes of TAO, and, thus, may be identified as novel therapeutic targets for this disease.

Additionally, we described the immune characteristics of BCR repertoires in TAO, revealing a significant increase in BCR diversity during active TAO.

Our observations shed light on the molecular and cellular basis of the inflammatory reaction observed in active TAO. By gaining a better understanding of the immune dysregulation in this condition, our study lays the groundwork for the development of more effective immunotherapy strategies in the future. With further validation and exploration of the underlying mechanisms, these discoveries hold the potential to contribute significantly to the advancement of targeted and personalized treatments for individuals with TAO.

## Supplementary Material

Supplementary Figures

Supplementary Tables

## References

[1] Smith TJ, Hegedüs L. Graves’ Disease. N Engl J Med. 2017; 376:185. 10.1056/NEJMc1614624 28076710

[2] Bahn RS. Graves’ ophthalmopathy. N Engl J Med. 2010; 362:726–38. 10.1056/NEJMra0905750 20181974 PMC3902010

[3] Khong JJ, McNab AA, Ebeling PR, Craig JE, Selva D. Pathogenesis of thyroid eye disease: review and update on molecular mechanisms. Br J Ophthalmol. 2016; 100:142–50. 10.1136/bjophthalmol-2015-307399 26567024

[4] Smith TJ, Janssen JA. Insulin-like Growth Factor-I Receptor and Thyroid-Associated Ophthalmopathy. Endocr Rev. 2019; 40:236–67. 10.1210/er.2018-00066 30215690 PMC6338478

[5] Kim DW, Taneja K, Hoang T, Santiago CP, McCulley TJ, Merbs SL, Mahoney NR, Blackshaw S, Rajaii F. Transcriptomic Profiling of Control and Thyroid-Associated Orbitopathy (TAO) Orbital Fat and TAO Orbital Fibroblasts Undergoing Adipogenesis. Invest Ophthalmol Vis Sci. 2021; 62:24. 10.1167/iovs.62.9.24 34269815 PMC8297424

[6] Wiersinga WM. Quality of life in Graves’ ophthalmopathy. Best Pract Res Clin Endocrinol Metab. 2012; 26:359–70. 10.1016/j.beem.2011.11.001 22632371

[7] Ponto KA, Merkesdal S, Hommel G, Pitz S, Pfeiffer N, Kahaly GJ. Public health relevance of Graves’ orbitopathy. J Clin Endocrinol Metab. 2013; 98:145–52. 10.1210/jc.2012-3119 23185037

[8] Taylor PN, Zhang L, Lee RW, Muller I, Ezra DG, Dayan CM, Kahaly GJ, Ludgate M. New insights into the pathogenesis and nonsurgical management of Graves orbitopathy. Nat Rev Endocrinol. 2020; 16:104–16. 10.1038/s41574-019-0305-4 31889140

[9] Wiersinga WM. Advances in treatment of active, moderate-to-severe Graves’ ophthalmopathy. Lancet Diabetes Endocrinol. 2017; 5:134–42. 10.1016/S2213-8587(16)30046-8 27346786

[10] Kim JW, Han SH, Son BJ, Rim TH, Keum KC, Yoon JS. Efficacy of combined orbital radiation and systemic steroids in the management of Graves’ orbitopathy. Graefes Arch Clin Exp Ophthalmol. 2016; 254:991–8. 10.1007/s00417-016-3280-7 26876240

[11] Oray M, Abu Samra K, Ebrahimiadib N, Meese H, Foster CS. Long-term side effects of glucocorticoids. Expert Opin Drug Saf. 2016; 15:457–65. 10.1517/14740338.2016.1140743 26789102

[12] Robertson DM, Buettner H, Gorman CA, Garrity JA, Fatourechi V, Bahn RS, Petersen IA, Stafford SL, Earle JD, Forbes GS, Kline RW, Bergstralh EJ, Offord KP, et al. Retinal microvascular abnormalities in patients treated with external radiation for graves ophthalmopathy. Arch Ophthalmol. 2003; 121:652–57. 10.1001/archopht.121.5.652 12742842

[13] McLachlan SM, Rapoport B. Breaking tolerance to thyroid antigens: changing concepts in thyroid autoimmunity. Endocr Rev. 2014; 35:59–105. 10.1210/er.2013-1055 24091783 PMC3895862

[14] Jansen K, Cevhertas L, Ma S, Satitsuksanoa P, Akdis M, van de Veen W. Regulatory B cells, A to Z. Allergy. 2021; 76:2699–715. 10.1111/all.14763 33544905

[15] Fang S, Huang Y, Zhong S, Li Y, Zhang Y, Li Y, Sun J, Liu X, Wang Y, Zhang S, Xu T, Sun X, Gu P, et al. Regulation of Orbital Fibrosis and Adipogenesis by Pathogenic Th17 Cells in Graves Orbitopathy. J Clin Endocrinol Metab. 2017; 102:4273–83. 10.1210/jc.2017-01349 28938397

[16] Fang S, Huang Y, Wang N, Zhang S, Zhong S, Li Y, Sun J, Liu X, Wang Y, Gu P, Li B, Zhou H, Fan X. Insights Into Local Orbital Immunity: Evidence for the Involvement of the Th17 Cell Pathway in Thyroid-Associated Ophthalmopathy. J Clin Endocrinol Metab. 2019; 104:1697–711. 10.1210/jc.2018-01626 30517642

[17] Antonelli A, Ferrari SM, Fallahi P, Frascerra S, Santini E, Franceschini SS, Ferrannini E. Monokine induced by interferon gamma (IFNgamma) (CXCL9) and IFNgamma inducible T-cell alpha-chemoattractant (CXCL11) involvement in Graves’ disease and ophthalmopathy: modulation by peroxisome proliferator-activated receptor-gamma agonists. J Clin Endocrinol Metab. 2009; 94:1803–9. 10.1210/jc.2008-2450 19276231

[18] Hwang CJ, Afifiyan N, Sand D, Naik V, Said J, Pollock SJ, Chen B, Phipps RP, Goldberg RA, Smith TJ, Douglas RS. Orbital fibroblasts from patients with thyroid-associated ophthalmopathy overexpress CD40: CD154 hyperinduces IL-6, IL-8, and MCP-1. Invest Ophthalmol Vis Sci. 2009; 50:2262–8. 10.1167/iovs.08-2328 19117935 PMC2752347

[19] Kuriyan AE, Woeller CF, O’Loughlin CW, Phipps RP, Feldon SE. Orbital fibroblasts from thyroid eye disease patients differ in proliferative and adipogenic responses depending on disease subtype. Invest Ophthalmol Vis Sci. 2013; 54:7370–7. 10.1167/iovs.13-12741 24135759 PMC3823547

[20] Łacheta D, Miśkiewicz P, Głuszko A, Nowicka G, Struga M, Kantor I, Poślednik KB, Mirza S, Szczepański MJ. Immunological Aspects of Graves’ Ophthalmopathy. Biomed Res Int. 2019; 2019:7453260. 10.1155/2019/7453260 31781640 PMC6875285

[21] Nagata K, Nakayama Y, Higaki K, Ochi M, Kanai K, Matsushita M, Kuwamoto S, Kato M, Murakami I, Iwasaki T, Nanba E, Kimura H, Hayashi K. Reactivation of persistent Epstein-Barr virus (EBV) causes secretion of thyrotropin receptor antibodies (TRAbs) in EBV-infected B lymphocytes with TRAbs on their surface. Autoimmunity. 2015; 48:328–35. 10.3109/08916934.2015.1022163 25759125

[22] Wang Y, Chen Z, Wang T, Guo H, Liu Y, Dang N, Hu S, Wu L, Zhang C, Ye K, Shi B. A novel CD4+ CTL subtype characterized by chemotaxis and inflammation is involved in the pathogenesis of Graves’ orbitopathy. Cell Mol Immunol. 2021; 18:735–45. 10.1038/s41423-020-00615-2 33514849 PMC8027210

[23] Li Z, Wang M, Tan J, Zhu L, Zeng P, Chen X, Xie L, Duan R, Chen B, Tao T, Wang R, Wang X, Su W. Single-cell RNA sequencing depicts the local cell landscape in thyroid-associated ophthalmopathy. Cell Rep Med. 2022; 3:100699. 10.1016/j.xcrm.2022.100699 35896115 PMC9418739

[24] Wu P, Lin B, Huang S, Meng J, Zhang F, Zhou M, Hei X, Ke Y, Yang H, Huang D. IL-11 Is Elevated and Drives the Profibrotic Phenotype Transition of Orbital Fibroblasts in Thyroid-Associated Ophthalmopathy. Front Endocrinol (Lausanne). 2022; 13:846106. 10.3389/fendo.2022.846106 35273577 PMC8902078

[25] Bartley GB, Gorman CA. Diagnostic criteria for Graves' ophthalmopathy. Am J Ophthalmol. 1995; 119:792–5. 10.1016/s0002-9394(14)72787-4 7785696

[26] Werner SC. Modification of the classification of the eye changes of Graves’ disease. Am J Ophthalmol. 1977; 83:725–7. 10.1016/0002-9394(77)90140-4 577380

[27] Dobin A, Davis CA, Schlesinger F, Drenkow J, Zaleski C, Jha S, Batut P, Chaisson M, Gingeras TR. STAR: ultrafast universal RNA-seq aligner. Bioinformatics. 2013; 29:15–21. 10.1093/bioinformatics/bts635 23104886 PMC3530905

[28] Satija R, Farrell JA, Gennert D, Schier AF, Regev A. Spatial reconstruction of single-cell gene expression data. Nat Biotechnol. 2015; 33:495–502. 10.1038/nbt.3192 25867923 PMC4430369

[29] Kuleshov MV, Jones MR, Rouillard AD, Fernandez NF, Duan Q, Wang Z, Koplev S, Jenkins SL, Jagodnik KM, Lachmann A, McDermott MG, Monteiro CD, Gundersen GW, Ma’ayan A. Enrichr: a comprehensive gene set enrichment analysis web server 2016 update. Nucleic Acids Res. 2016; 44:W90–7. 10.1093/nar/gkw377 27141961 PMC4987924

[30] Liberzon A, Birger C, Thorvaldsdóttir H, Ghandi M, Mesirov JP, Tamayo P. The Molecular Signatures Database (MSigDB) hallmark gene set collection. Cell Syst. 2015; 1:417–25. 10.1016/j.cels.2015.12.004 26771021 PMC4707969

[31] Tirosh I, Venteicher AS, Hebert C, Escalante LE, Patel AP, Yizhak K, Fisher JM, Rodman C, Mount C, Filbin MG, Neftel C, Desai N, Nyman J, et al. Single-cell RNA-seq supports a developmental hierarchy in human oligodendroglioma. Nature. 2016; 539:309–13. 10.1038/nature20123 27806376 PMC5465819

[32] Mourits MP, Prummel MF, Wiersinga WM, Koornneef L. Clinical activity score as a guide in the management of patients with Graves’ ophthalmopathy. Clin Endocrinol (Oxf). 1997; 47:9–14. 10.1046/j.1365-2265.1997.2331047.x 9302365

[33] Hendricks J, Bos NA, Kroese FG. Heterogeneity of Memory Marginal Zone B Cells. Crit Rev Immunol. 2018; 38:145–58. 10.1615/CritRevImmunol.2018024985 29887727 PMC5989013

[34] King HW, Orban N, Riches JC, Clear AJ, Warnes G, Teichmann SA, James LK. Single-cell analysis of human B cell maturation predicts how antibody class switching shapes selection dynamics. Sci Immunol. 2021; 6:eabe6291. 10.1126/sciimmunol.abe6291 33579751

[35] Catalán D, Mansilla MA, Ferrier A, Soto L, Oleinika K, Aguillón JC, Aravena O. Immunosuppressive Mechanisms of Regulatory B Cells. Front Immunol. 2021; 12:611795. 10.3389/fimmu.2021.611795 33995344 PMC8118522

[36] Kojetin DJ, Burris TP. REV-ERB and ROR nuclear receptors as drug targets. Nat Rev Drug Discov. 2014; 13:197–216. 10.1038/nrd4100 24577401 PMC4865262

[37] Li N, Wang N, He W, Feng Y, Qiu Q, Qiu H, Zheng L, Yin Y, Wang B, Sun Y, Pan C, Sample KM, Huang J, et al. The suppressive functions of Rora in B lineage cell proliferation and BCR/ABL1-induced B-ALL pathogenesis. Int J Biol Sci. 2022; 18:2277–91. 10.7150/ijbs.68939 35414788 PMC8990459

[38] Balamurugan K, Sterneck E. The many faces of C/EBPδ and their relevance for inflammation and cancer. Int J Biol Sci. 2013; 9:917–33. 10.7150/ijbs.7224 24155666 PMC3805898

[39] Wang YH, Tsai DY, Ko YA, Yang TT, Lin IY, Hung KH, Lin KI. Blimp-1 Contributes to the Development and Function of Regulatory B Cells. Front Immunol. 2019; 10:1909. 10.3389/fimmu.2019.01909 31474988 PMC6702260

[40] Ubieta K, Garcia M, Grötsch B, Uebe S, Weber GF, Stein M, Ekici A, Schett G, Mielenz D, Bozec A. Fra-2 regulates B cell development by enhancing IRF4 and Foxo1 transcription. J Exp Med. 2017; 214:2059–71. 10.1084/jem.20160514 28566276 PMC5502419

[41] Mishra S, Srinivasan S, Ma C, Zhang N. CD8+ Regulatory T Cell - A Mystery to Be Revealed. Front Immunol. 2021; 12:708874. 10.3389/fimmu.2021.708874 34484208 PMC8416339

[42] Zhang J, Marotel M, Fauteux-Daniel S, Mathieu AL, Viel S, Marçais A, Walzer T. T-bet and Eomes govern differentiation and function of mouse and human NK cells and ILC1. Eur J Immunol. 2018; 48:738–50. 10.1002/eji.201747299 29424438

[43] Malissen B, Tamoutounour S, Henri S. The origins and functions of dendritic cells and macrophages in the skin. Nat Rev Immunol. 2014; 14:417–28. 10.1038/nri3683 24854591

[44] Gay NJ, Symmons MF, Gangloff M, Bryant CE. Assembly and localization of Toll-like receptor signalling complexes. Nat Rev Immunol. 2014; 14:546–58. 10.1038/nri3713 25060580

[45] Elizondo DM, Brandy NZD, da Silva RLL, Haddock NL, Kacsinta AD, de Moura TR, Lipscomb MW. Allograft Inflammatory Factor-1 Governs Hematopoietic Stem Cell Differentiation Into cDC1 and Monocyte-Derived Dendritic Cells Through IRF8 and RelB *in vitro*. Front Immunol. 2019; 10:173. 10.3389/fimmu.2019.00173 30800127 PMC6375893

[46] Elizondo DM, Andargie TE, Yang D, Kacsinta AD, Lipscomb MW. Inhibition of Allograft Inflammatory Factor-1 in Dendritic Cells Restrains CD4+ T Cell Effector Responses and Induces CD25+Foxp3+ T Regulatory Subsets. Front Immunol. 2017; 8:1502. 10.3389/fimmu.2017.01502 29167673 PMC5682305

[47] Manz BN, Tan YX, Courtney AH, Rutaganira F, Palmer E, Shokat KM, Weiss A. Small molecule inhibition of Csk alters affinity recognition by T cells. Elife. 2015; 4:e08088. 10.7554/eLife.08088 26302204 PMC4568592

[48] Vermot A, Petit-Härtlein I, Smith SM, Fieschi F. NADPH Oxidases (NOX): An Overview from Discovery, Molecular Mechanisms to Physiology and Pathology. Antioxidants (Basel). 2021; 10:890. 10.3390/antiox10060890 34205998 PMC8228183

[49] Lanzolla G, Marcocci C, Marinò M. Oxidative Stress in Graves Disease and Graves Orbitopathy. Eur Thyroid J. 2020 (Suppl 1); 9:40–50. 10.1159/000509615 33511084 PMC7802440

[50] Yang M, Du C, Wang Y, Liu J. CD19+CD24hiCD38hi regulatory B cells are associated with insulin resistance in type I Hashimoto's thyroiditis in Chinese females. Exp Ther Med. 2017; 14:3887–93. 10.3892/etm.2017.4925 29042997 PMC5639318

[51] Ma K, Du W, Wang X, Yuan S, Cai X, Liu D, Li J, Lu L. Multiple Functions of B Cells in the Pathogenesis of Systemic Lupus Erythematosus. Int J Mol Sci. 2019; 20:6021. 10.3390/ijms20236021 31795353 PMC6929160

[52] Ummarino D. Rheumatoid arthritis: Defective IL-10-producing Breg cells. Nat Rev Rheumatol. 2017; 13:132. 10.1038/nrrheum.2017.10 28148914

[53] Ding YG, Chen G, Li Q, Wen XF, Wei L, Yang HS. Frequency of IL-10-producing regulatory B cells associated with disease activity in thyroid-associated orbitopathy. Int J Ophthalmol. 2018; 11:1458–62. 10.18240/ijo.2018.09.05 30225218 PMC6133891

[54] Zha B, Wang L, Liu X, Liu J, Chen Z, Xu J, Sheng L, Li Y, Chu Y. Decrease in proportion of CD19+ CD24(hi) CD27+ B cells and impairment of their suppressive function in Graves’ disease. PLoS One. 2012; 7:e49835. 10.1371/journal.pone.0049835 23189166 PMC3506658

[55] Chen G, Ding Y, Li Q, Li Y, Wen X, Ji X, Bi S, Chen J, Xu J, Chen R, Ye H, Wei L, Yang H. Defective Regulatory B Cells Are Associated With Thyroid-Associated Ophthalmopathy. J Clin Endocrinol Metab. 2019; 104:4067–77. 10.1210/jc.2018-01812 30888403

[56] Wojciechowska-Durczynska K, Wieczorek-Szukala K, Stefanski B, Zygmunt A, Stepniak J, Karbownik-Lewinska M, Lewinski A. Percentage of Myeloid Dendritic Cells in Peripheral Venous Blood Is Negatively Related to Incidence of Graves’ Orbitopathy. Mediators Inflamm. 2021; 2021:8896055. 10.1155/2021/8896055 33574732 PMC7857924

[57] Jianan Xu HYRC. Distribution of monocyte subtypes in peripheral blood of patients with thyroid associated ophthalmopathy. Chin J Exp Ophthalmol. 2020; 38.

[58] Narasimhan PB, Marcovecchio P, Hamers AAJ, Hedrick CC. Nonclassical Monocytes in Health and Disease. Annu Rev Immunol. 2019; 37:439–56. 10.1146/annurev-immunol-042617-053119 31026415

[59] Mauri C, Bosma A. Immune regulatory function of B cells. Annu Rev Immunol. 2012; 30:221–41. 10.1146/annurev-immunol-020711-074934 22224776

[60] Tsubata T. Inhibitory B cell co-receptors and autoimmune diseases. Immunol Med. 2019; 42:108–16. 10.1080/25785826.2019.1660038 31532707

[61] Ning Z, Liu K, Xiong H. Roles of BTLA in Immunity and Immune Disorders. Front Immunol. 2021; 12:654960. 10.3389/fimmu.2021.654960 33859648 PMC8043046

[62] Thibult ML, Rivals JP, Mamessier E, Gertner-Dardenne J, Pastor S, Speiser DE, Derré L, Olive D. CpG-ODN-induced sustained expression of BTLA mediating selective inhibition of human B cells. J Mol Med (Berl). 2013; 91:195–205. 10.1007/s00109-012-0943-7 22903545

[63] Ramadoss NS, Robinson WH. Characterizing the BCR repertoire in immune-mediated diseases. Nat Rev Rheumatol. 2020; 16:7–8. 10.1038/s41584-019-0339-y 31780792 PMC7194096

[64] Hoehn KB, Fowler A, Lunter G, Pybus OG. The Diversity and Molecular Evolution of B-Cell Receptors during Infection. Mol Biol Evol. 2016; 33:1147–57. 10.1093/molbev/msw015 26802217 PMC4839220

