# Supplementary material for: Single-cell BCR and transcriptome analysis reveals peripheral immune signatures in patients with thyroid-associated ophthalmopathy
Source: Aging (Albany NY). 2024 May 9;16(9):8217–45. doi: 10.18632/aging.205814 (PMC11132005; doi:10.18632/aging.205814)
Supplement: Supplementary Figures [file aging-16-205814-s001.pdf]

SUPPLEMENTARY FIGURES

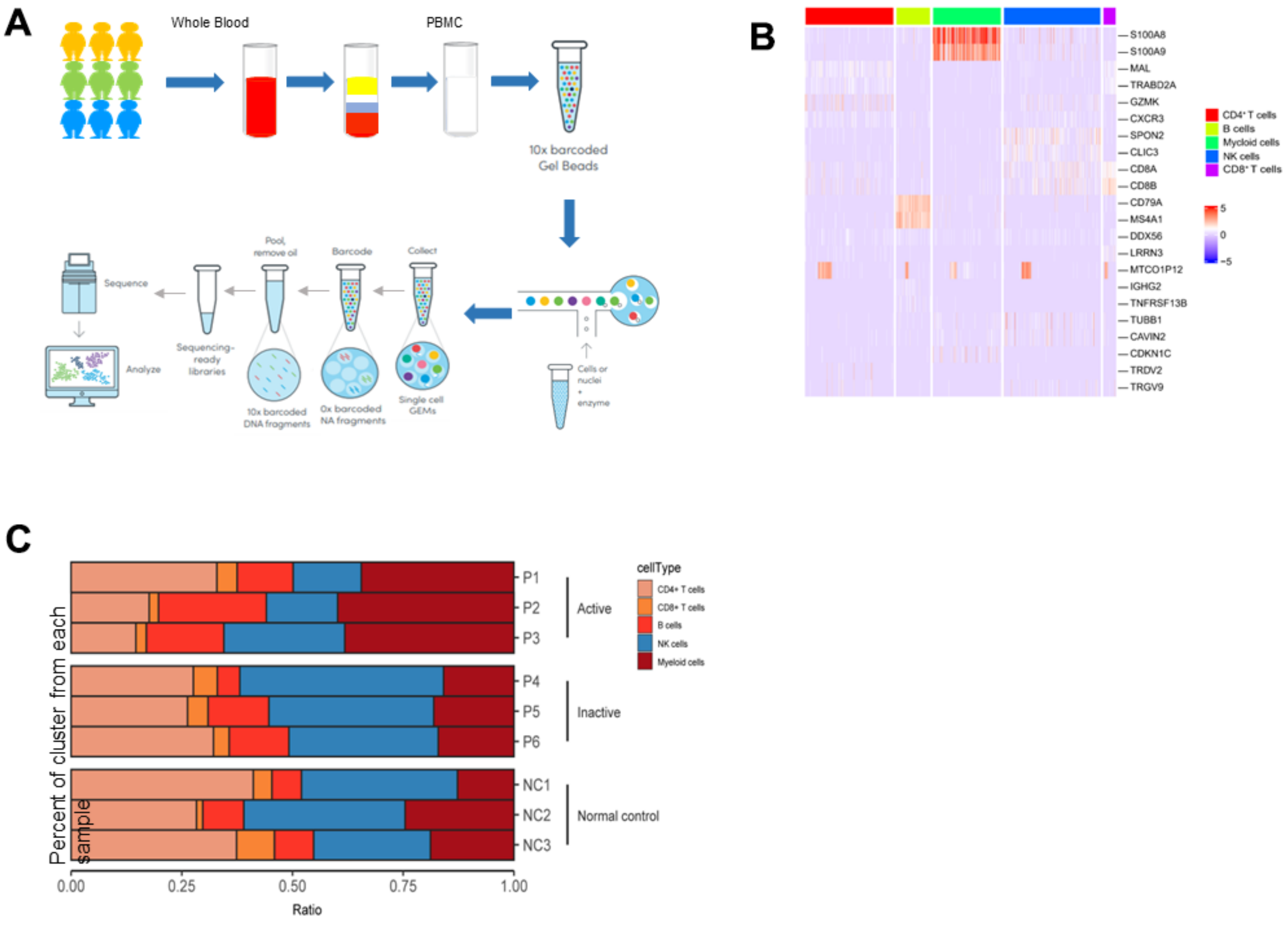

Supplementary Figure 1. The supplementary figures for Figure 1. (**A**) Flow chart of the overall study. (**B**) Heatmap of top 2 DEGs in each cell populations. Key genes for each cell type are listed on the right margin. Expression values are scaled between minimum and maximum expression for each gene across all clusters. (**C**) Prevalence of each cell type (columns) across all samples from all patients studied (rows).

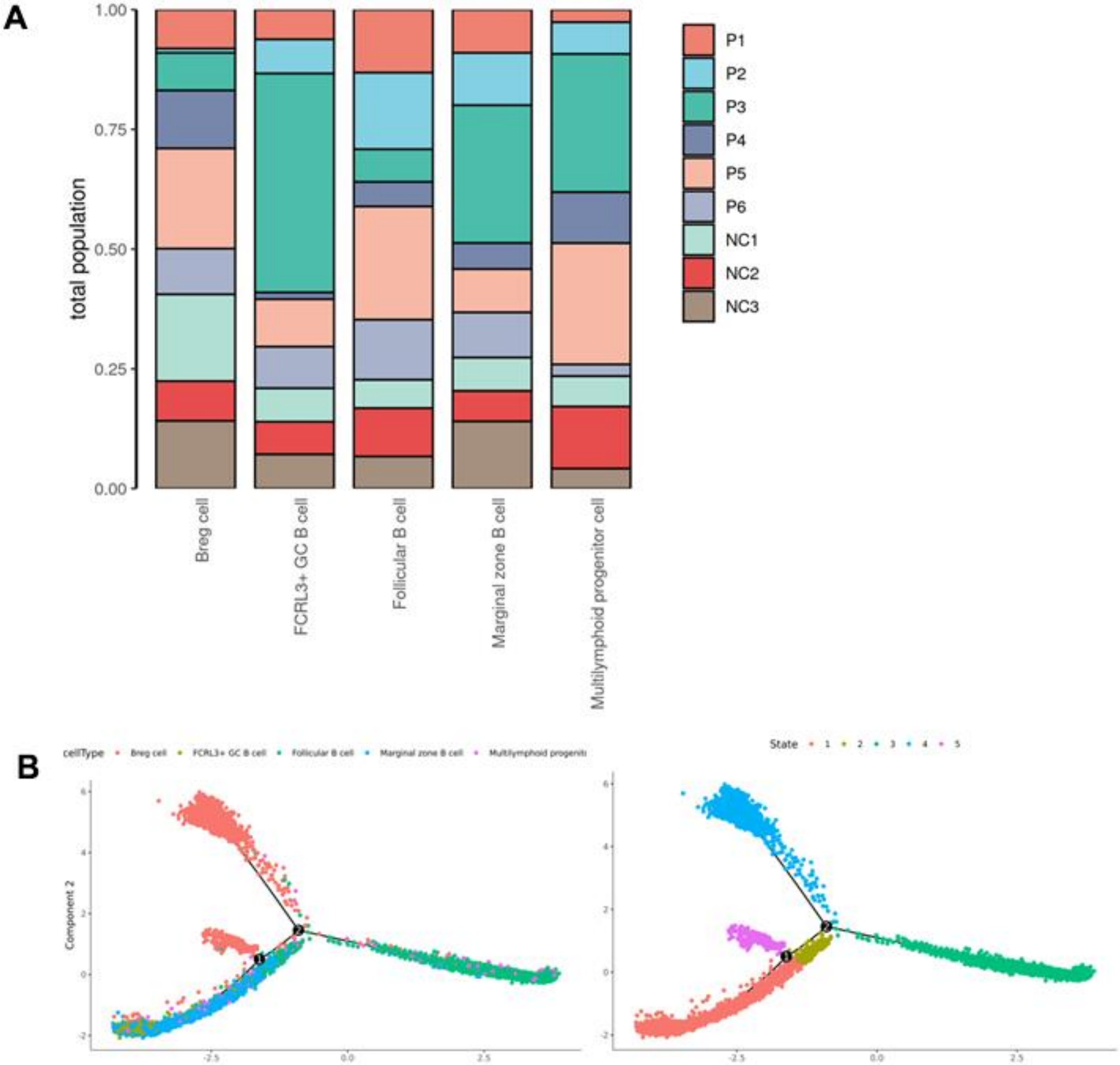

Supplementary Figure 2. The supplementary figures for Figure 2. (**A**) Representation of each sample within each B cell cluster. (**B**) The trajectory analysis of B cell subsets in all samples.

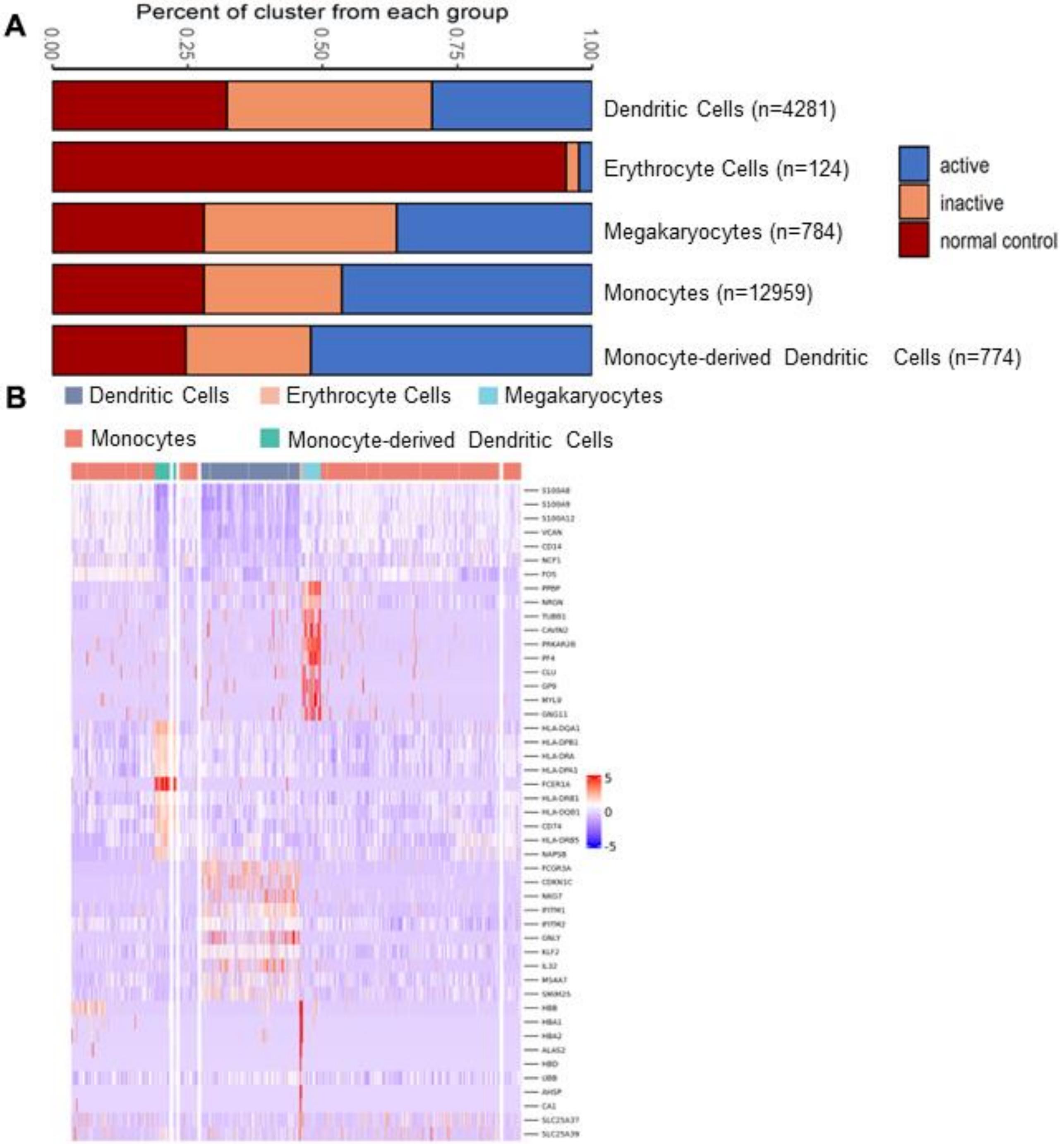

Supplementary Figure 3. The supplementary figures for Figure 4. (**A**) Representation of each sample within each myeloid cluster. (**B**) Heatmap of DEGs for individual myeloid populations.

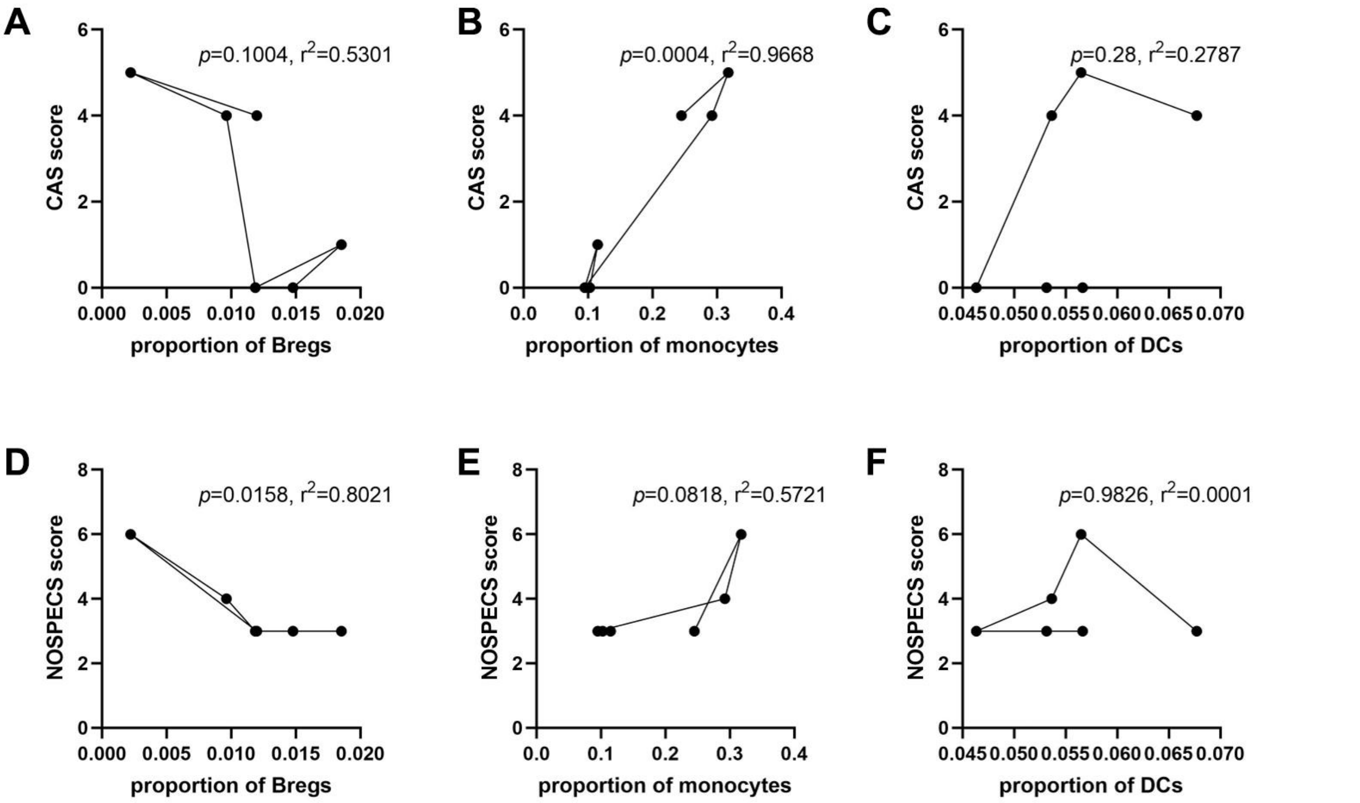

Supplementary Figure 4. Correlation analyses between the percentage of Bregs, monocytes, DCs and clinical indicators of TAO. The correlation analyses between the proportion of (**A**) Bregs, (**B**) monocytes, (**C**) DCs and disease activity of TAO. The correlation analyses between the proportion of (**D**) Bregs, (**E**) monocytes, (**F**) DCs and disease severity of TAO.

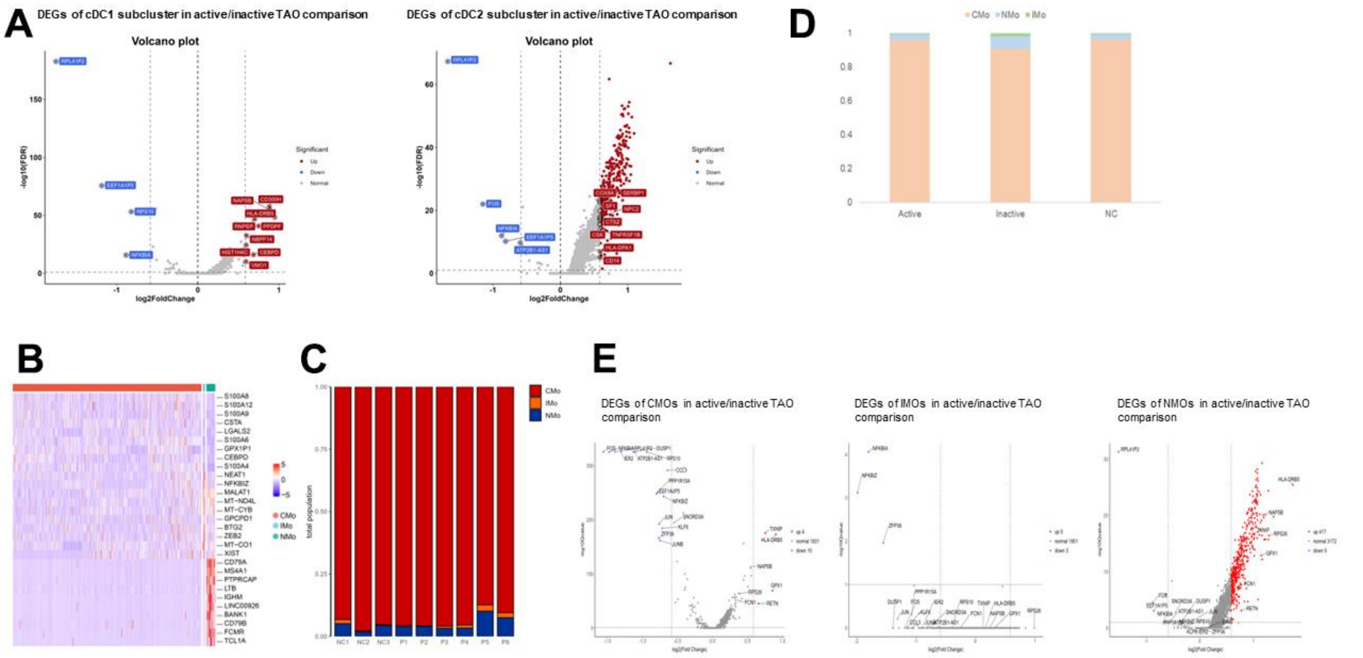

Supplementary Figure 5. The supplementary figures for Figure 5 and 6. (**A**) Volcano plots showing DEGs of cDC1 subcluster and cDC2 subcluster in active/inactive TAO comparison. (**B**) Heatmap showing scaled expression of DEGs for monocyte subsets. (**C**) Prevalence of CMOs, NMOs, and IMOs across all samples from all individuals studied. (**D**) Histogram showing the relative proportion of monocyte subsets from active TAO, inactive TAO and NCs. (**E**) Volcano plots showing DEGs of CMOs, IMOs and NMOs in active/inactive TAO comparison.
