# Supplementary material for: Single-cell BCR and transcriptome analysis reveals peripheral immune signatures in patients with thyroid-associated ophthalmopathy
Source: Aging (Albany NY). 2024 May 9;16(9):8217–45. doi: 10.18632/aging.205814 (PMC11132005; doi:10.18632/aging.205814)
Supplement: Supplementary Tables [file aging-16-205814-s002.pdf]

SUPPLEMENTARY TABLES

Supplementary Table 1. Number of filtered cells and number of median genes for each cell.

Patient	Number of filtered cells	Median genes/cell
P 1	8161	1802
P 2	4727	1684
P 3	10163	1806
P 4	12521	1433
P 5	14050	1406
P 6	8226	1329
NC 1	12051	1486
NC 2	12360	1453
NC 3	10748	1587

Supplementary Table 2. The annotation of 17 cell subpopulations.

Cluster ID	Cell Annotation Type
0	myeloid cells
1	T cells, CD4+
2	T cells, CD4+
3	NK cells
4	NK cells
5	B cells
6	NK cells
7	T cells, CD8+
8	B cells
9	NK cells
10	myeloid cells
11	T cells, CD4+
12	T cells, CD4+
13	myeloid cells
14	NK cells
15	NA
16	NK cells

Supplementary Table 3. The number and proportion of each cell type for each sample.

	Active			Inactive			Normal Ctrl		
**sample ID**	P1	P2	P3	P4	P5	P6	NC1	NC2	NC3
**CD4+ T cells**	2530	796	1354	3189	3365	2350	4548	2561	3787
**CD8+ T cells**	351	95	217	625	592	259	466	127	865
**NK cells**	1180	726	2525	5313	4762	2463	3897	3293	2674
**Myeloid cells**	2648	1797	3533	1836	2317	1251	1408	2223	1909
**B cells**	972	1100	1618	586	1760	987	736	839	895
**total cell numbers**	7681	4514	9247	11549	12796	7310	11055	9043	10130
**proportion of CD4+ T cells**	0.33	0.18	0.15	0.28	0.26	0.32	0.41	0.28	0.37
**proportion of CD8+ T cells**	0.05	0.02	0.02	0.05	0.05	0.04	0.04	0.01	0.09
**proportion of NK cells**	0.15	0.16	0.27	0.46	0.37	0.34	0.35	0.36	0.26
**proportion of myeloid cells**	0.34	0.40	0.38	0.16	0.18	0.17	0.13	0.25	0.19
**proportion of B cells**	0.13	0.24	0.17	0.05	0.14	0.14	0.07	0.09	0.09

Supplementary Table 4. The clinical relevance of quantitative changes of Bregs, DCs, and monocytes in TAO.

		**Active**		**Inactive**		
**sample ID**	**P1**	**P2**	**P3**	**P4**	**P5**	**P6**
**number of Bregs**	92	10	89	137	237	108
**number of monocytes**	1880	1433	2703	1095	1468	750
**number of DC**	520	255	496	535	680	414
**total cell numbers**	7681	4514	9247	11549	12796	7310
**proportion of Bregs**	0.011978	0.002215	0.009625	0.011862	0.018521	0.0147743
**proportion of monocytes**	0.24476	0.317457	0.292311	0.094813	0.114723	0.1025992
**proportion of DC**	0.0677	0.056491	0.053639	0.046324	0.053142	0.0566347
**CAS score**	4	5	4	0	1	0
**NOSPECS score**	3	6	4	3	3	3
